# Comparative Genomics and Transcriptomics Analyses Reveal Divergent Lifestyle Features of Nematode Endoparasitic Fungus *Hirsutella minnesotensis*

**DOI:** 10.1093/gbe/evu241

**Published:** 2014-10-30

**Authors:** Yiling Lai, Keke Liu, Xinyu Zhang, Xiaoling Zhang, Kuan Li, Niuniu Wang, Chi Shu, Yunpeng Wu, Chengshu Wang, Kathryn E. Bushley, Meichun Xiang, Xingzhong Liu

**Affiliations:** ^1^State Key Laboratory of Mycology, Institute of Microbiology, Chinese Academy of Sciences, Beijing, China; ^2^University of Chinese Academy of Sciences, Beijing, China; ^3^Key Laboratory of Insect Developmental and Evolutionary Biology, Institute of Plant Physiology and Ecology, Shanghai Institutes for Biological Sciences, Chinese Academy of Sciences, Shanghai, China; ^4^College of Biological Sciences, University of Minnesota

**Keywords:** nematode endoparasitic fungus, life strategy, parasitism mechanism, comparative genomics, transcriptome

## Abstract

*Hirsutella minnesotensis* [Ophiocordycipitaceae (Hypocreales, Ascomycota)] is a dominant endoparasitic fungus by using conidia that adhere to and penetrate the secondary stage juveniles of soybean cyst nematode. Its genome was de novo sequenced and compared with five entomopathogenic fungi in the Hypocreales and three nematode-trapping fungi in the Orbiliales (Ascomycota). The genome of *H. minnesotensis* is 51.4 Mb and encodes 12,702 genes enriched with transposable elements up to 32%. Phylogenomic analysis revealed that *H. minnesotensis* was diverged from entomopathogenic fungi in Hypocreales. Genome of *H. minnesotensis* is similar to those of entomopathogenic fungi to have fewer genes encoding lectins for adhesion and glycoside hydrolases for cellulose degradation, but is different from those of nematode-trapping fungi to possess more genes for protein degradation, signal transduction, and secondary metabolism. Those results indicate that *H. minnesotensis* has evolved different mechanism for nematode endoparasitism compared with nematode-trapping fungi. Transcriptomics analyses for the time-scale parasitism revealed the upregulations of lectins, secreted proteases and the genes for biosynthesis of secondary metabolites that could be putatively involved in host surface adhesion, cuticle degradation, and host manipulation. Genome and transcriptome analyses provided comprehensive understanding of the evolution and lifestyle of nematode endoparasitism.

## Introduction

**Fig. 1.—:**
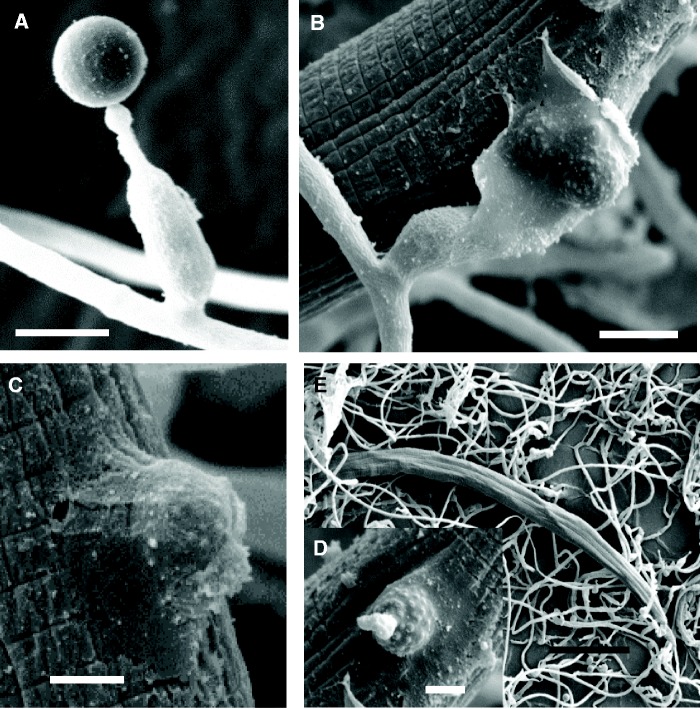
Conidia and the infection stages of *Hirsutella minnesotensis* on SCN. (*A*) Conidium of *H. minnesotensis*. (*B*, *C*) A conidium adhered to the cuticle of a passby nematode by secretion of adhesive substances. (*D*) The cuticle of nematode was degraded and penetrated by an adhesive conidium. (*E*) The fungus grew in the body of the nematode. Bars: 5.0 μm for (*A*) and (*B*), 2.5 μm for (*C*) and (*D*), and 50.0 μm for (*E*).

Nematophagous fungi are a diverse group of carnivorous fungal species that use their specific mycelia structures or conidia to capture the vermiform nematodes, or use their hypha tips to parasitize the eggs and cysts of nematodes, or produce toxins to attack nematodes (Liu et al. 2009). Two main groups of nematophagous fungi are known as nematode-trapping fungi invading nematode by producing specific trapping devices, and nematode endoparasitic fungi invading nematode by using adhesive or digesting conidia. *Hirsutella minnesotensis* (Ophiocordycipitaceae, Hypocreales, Ascomycota) is the representative of nematode endoparasitic fungi. This species mainly invades the secondary stage juveniles of cyst nematodes by producing conidia that adhere to, penetrate cuticle, and eventually kill the nematode (fig. 1). The fungus consumes the nematode body content and finally grows out from the cadaver to produce new conidia for the next infection cycle (Sturhan and Schneider 1980; Liu and Chen 2000).

*Hirsutella minnesotensis* has been mainly isolated from soybean cyst nematode (SCN) *Heterodera glycines* in the United States and China (Liu and Chen 2000; Ma et al. 2005); whereas, on agar plates in laboratory conditions, it can parasitize 15 different nematode species belonging to 12 genera, including plant-parasitic, entomopathogenic, fungal-feeding, and bacteria-feeding nematodes (Liu and Chen 2001). As endoparasite, *H. minnesotensis* is the dominant species on SCNs in China (Liu and Chen 2000; Ma et al. 2005). High percentages of nematodes parasitized in field soils and responses to the suppressiveness of SCN in soybean monoculture system indicate that *H. minnesotensis* is crucial factor in regulation nematode populations in nature. Field experiments and greenhouse studies also suggest that *H. minnesotensis* has great potential as biological control agent (Lackey et al. 1993; Liu and Chen 2001, 2005; Chen and Liu 2005; Mennan et al. 2006).

*Hirsutella minnesotensis* and *Hirsutella rhossiliensis* are the two closely related species detected from cyst nematodes and are the members of the Hypocreales. Hypocreales includes many species that also tend to associated with animals (Sung et al. 2008). These species are common and/or important entomopathogenic fungi, such as *Beauvaria bassiana* and *Metarhizium robertsii* for insect biocontrol (Gao et al. 2011; Xiao et al. 2012), *Cordyceps militaris* and *Ophiocordyceps sinensis* (anamorph: *Hirsutella sinensis*) for medicinal application (Zheng et al. 2011; Hu et al. 2013), and *Tolypocladium inflatum*, which produces the immunosuppressant drug cyclosporine (Bushley et al. 2013). However, *H. minnesotensis* and *H. rhossiliensis* described to date are unique in their capacity to attack nematodes, suggesting a relatively recent, independent origin of the nematode endoparasitic lifestyle.

Unlike entomopathogenic fungi which usually attach their spores to the external body surface of insects, or trapping fungi which capture nematodes by various trap devices developed from hyphae, *H. minnesotensis* and *H. rhossiliensis* produce adhesive spores to parasitize the nematode; however, the molecular basis of this fascinating biological phenomenon remains poorly understood. Only two genes encoding a serine protease were identified and emerged as a useful factor to penetrate the host’s cuticle in *H. rhossiliensis* (Wang et al. 2007, 2009).

Comparative genomic research has become an essential approach for better understanding of fungal evolution, biology, and interactions with hosts and secondary metabolism. Although trapping fungi in the Orbiliales are phylogenetically distant from entomopathogenic and endoparasitic fungi in the Hypocreales, their origin, evolution, trap formation, and trapping mechanism have been elucidated based on the genomes of *Arthrobotrys oligospora* forming adhesive networks (Yang et al. 2011), *Monacrosporium haptotylum* forming adhesive knobs (Meerupati et al. 2013), and *Drechslerella stenobrocha* forming constricting rings (Liu et al. 2014). Genomic studies of fungi in the Hypocreales have revealed that gene duplication has driven host range and pathogenic capabilities of *Me. robertsii* and *B. bassiana* (Gao et al. 2011; Xiao et al. 2012). Inbreeding in *O. sinensis* has caused massive proliferation of retrotransposons, providing a tradeoff between the advantages of increased genetic variation independent of sexual recombination and deletion of genes dispensable for its specialized pathogenic lifestyle (Xiao et al. 2012). Similarly, the current known mycotoxin genes are lacking in the medicinal fungus *C. militaris* (Zheng et al. 2011) and *T. inflatum* has developed the cyclosporine biosynthetic gene cluster (Bushley et al. 2013).

Although a great deal is known about the ecology of nematode endoparasitism and the biology of this fungus in laboratory, relatively little is known about the mechanism of endoparasitic lifestyle or how it evolved the ability to infect nematode. To address these questions, the whole genome of *H. minnesotensis* was sequenced and compared with trapping fungi in Orbiliales and other related entomopathogenic fungi in the Hypocreales. The transcriptome during the infection process of *Caenorhabditis elegans* by *H. minnesotensis* was also analyzed. The genome sequence and transcriptome analyses allow a comparison of genes involved in nematode endoparasitism across a range of evolutionary distances and elucidate an understanding of how the fungus evolved to associate with nematodes.

## Materials and Methods

### Fungal Strains

*Hirsutella minnesotensis* strain 3608 was selected for genome sequencing. *Hirsutella minnesotensis* was originally isolated from SCN (*H. glycines* Ichinohe) in Minnesota, USA (Chen et al. 2000), and strain 3608 was isolated from SCN in Jiamusi, Heilongjiang Province, China. This strain shows commercial potential for biological controls of SCNs. Twenty single spores were isolated from this fungus culture on corn meal agar (CMA; BD) plate and cultures of each spore were tested for their parasitic ability on SCNs. The single spore culture with the highest parasitic rate was selected and grown in potato dextrose agar (BD) plate for 10 days. Genomic DNA was prepared using the CTAB/SDS/Proteinase K method (Moller et al. 1992). A Quanta 200 (FEI, Netherlands) scanning electron microscope was used to observe the parasitism of *H. minnesotensis* on SCNs (Luo et al. 2004).

### Genome Sequencing and Assembly

Whole genome shotgun sequencing of *H. minnesotensis* strain 3608 was performed using the Illumina next generation sequencing technology. DNA libraries with 170-, 500-bp, 2-, and 5-kb inserts were constructed and sequenced with the Illumina Genome Analyzer at the Beijing Genomics Institute at Shenzhen. Quality control of reads was realized by FastQC. Low quality reads, reads with a certain proportion of *N* (10%), adapter contamination (15-bp overlap between adapter and reads), and duplication contamination were removed to obtain clean reads before assembly. The Kmer value of 51 was used for the genome assembly. The genome was sequenced to 128.3-fold coverage and assembled using SOAPdenovo (Li et al. 2009).

### Genome Annotation

To maximize accuracy, EvidenceModeler algorithms (Haas et al. 2008) were used to predict the gene structures of *H. minnesotensis*, using the sequenced genome of *Fusarium graminearum* as the reference. PseudoPipe with default settings was used to identify pseudogenes (Zhang et al. 2006). All predicted gene models were annotated by InterproScan analysis (http://www.ebi.ac.uk/Tools/pfa/iprscan/). SignalP 3.0 (http://www.cbs.dtu.dk/services/SignalP/) and TargetP (http://www.cbs.dtu.dk/services/TargetP/) were used to predict potential secreted proteins.

### Transposable Elements and Repeat-Induced Point Mutation Analysis

Simple repeat rate has been estimated by Basic Local Alignment Search Tool (BLAST) against the RepeatMasker library (http://www.repeatmasker.org/). The REPET pipeline (Quesneville et al. 2005) was used to predict transposable elements (TEs) de novo. The TEdenovo program was used to detect TEs and group them in families by BLASTER using BLAST with the Repbase database (Quesneville et al. 2005). Genomic regions containing more than 60% TEs were identified using sliding genomic windows of 50 kb using an in-house script. RIP index was determined by program RIPCAL (Hane and Oliver 2008). To analyze the effect of proximity of a gene to a repeat region, the RIP index (TpA/ApT) of these genes was calculated as a function of distance to the repeat sequences (Ohm et al. 2012). RIP index (TpA/ApT) exceeding 0.89 was considered as significant (Hane and Oliver 2008). All of the TEs were taken into account.

### Orthology and Phylogenomic Analysis

OrthoMCL (http://www.orthomcl.org/) was used to construct gene families for the *H. minnesotensis* and 14 additional genomes (supplementary table S1, Supplementary Material online). Sequences with an *e* value cutoff of 1e-5 and at least 40% identity over 60% coverage were considered orthologs (Liu et al. 2014). To infer phylogenomic relationships, protein alignments were generated using MUSCLE (Edgar 2004) for each of 898 single-copy orthologs. Nonconserved regions in each multiple alignment were removed using an in-house script. The conserved region of each single copy ortholog was concatenated into one sequence and the maximum phylogenomic tree of the 16 species was created by using RAxML with the best model LG+I+G identified using ProtTest (Stamatakis 2006). The divergence time between species was estimated by the Penalized Likelihood method (Sanderson 2002) with r8s (Taylor and Berbee 2006) version 1.8 (http://loco.biosci.arizona.edu/r8s/) calibrated against the origin of Ascomycota at 500–600 Ma (Lucking et al. 2009).

### Proteases

The families of proteases were classified by full-length BLASTp against the MEROPS peptidase database (*E* < 1 × 10^−^^4^) (http://merops.sanger.ac.uk/). False positives were eliminated following unsuccessful searches against peptidase units and peptidase domains of MEROPS (*E* < 1 × 10^−^^4^) and the Pfam databases, respectively. Prediction of putative secreted proteases was then identified using a combination of the SignalP 3.0 and TargetP 1.1 servers.

### Secondary Metabolism

The AntiSMASH pipeline (http://antismash.secondarymetabolites.org/) with Hidden Markov Model (HMM) signatures was used to identify and annotate putative polyketide synthase (PKS), nonribosomal peptide synthetase (NRPS), and terpene synthase (TPS) genes and gene clusters, and to predict the PKS and NRPS domain architecture in all 16 genomes. Sequences were aligned with MUSCLE. RAxML (Stamatakis 2006) was used to construct a maximal-likelihood phylogenetic tree with the LG+I+G+F model identified using ProtTest. The gene order and conservation of clusters were manually inspected with the gene cluster alignment results from AntiSMASH website. A difference of 50% in the number of accessory genes between *H. minnesotensis* and other fungi was considered significantly different (Martinez et al. 2012).

### Other Protein Families

Putative enzymes involved in carbohydrate utilization were identified by a combination of BLASTp and HMMer searches against the carbohydrate-active enzymes database (http://www.cazy.org/). Whole genome blast searches were conducted against protein sequences in the pathogen–host interaction database (http://www.phi-base.org/) (*E* < 1 ×10^−^^5^). Transporters were classified based on the Transport Classification Database (http://www.tcdb.org/tcdb/). The cytochrome P450s were identified according to Dr Nelson’s P450 database (http://drnelson.utmem.edu/CytochromeP450.html). Lipases were classified according to BLASTp (*E* < 1 ×10^−^^5^) results obtained against the Lipase Engineering Database (http://www.led.uni-stuttgart.de/). Additionally, G-protein-coupled receptors (GPCRs), protein kinases, and transcription factors were classified by BLASTp against GPCDB sequences containing seven transmembrane helices (http://www.cbs.dtu.dk/services/TMHMM/), KinBase (http://kinase.com/), and Fungal Transcription Factor Database (http://ftfd.snu.ac.kr/), respectively. CAFÉ (De Bie et al. 2006) was used to analyze the evolution of the protein family size (expansion or contraction). Unpaired two-tailed Student’s *t*-test was conducted to compare the difference of protein family sizes between nematophagous fungi and other fungi. A *P* value of less than 0.05 was considered to be significant.

### Transcriptome Analysis

Wild-type *C. elegans* Bristol strain N2 was routinely cultured on *Escherichia coli* strain OP50 and maintained following the protocol described by Brenner (1974), with minor modifications. The gravid adult nematodes were collected on a 35-μm-aperture sieve, washed with sterile water, and lysed in a sodium hypochlorite solution to isolate the eggs. The eggs were hatched overnight at 22 °C to obtain the first-stage larvae (L1). The second-stage larvae (L2) of *C. elegans* were then obtained as described by Xie et al. (2010). *Hirsutella minnesotensis* strain 3608 was cultured on CMA plates for 10 days to produce a large amount of conidia and then cocultured with 6,000 L2s for each plate. After 8, 16, and 36 h at 25 °C, the TRIzol method (Rio et al. 2010) was used to extract total RNA, which was then reverse transcribed into double-stranded cDNA and sequenced using the Illumina technique. Five million reads were obtained using Paired-End (PE100) sequencing for each transcript. Functions of the expressed genes were predicted based on Funcat, GO, and Pfam databases. The program maSigPro was used to enrich the genes expressed in different patterns (Conesa et al. 2006). The program NOISeq (Mortazavi et al. 2008) was used to calculate the probability value of each differently expressed gene.

### Nematode Bioassays

To test the effect of lectins on parasitism, the second juveniles of SCNs (J2) were treated with different lectins at the concentration of 100 μg/ml, including mannose binding lectin, concanavalin A lectin, Ricin B lectin, and lectin from *Helix pomatia*, and their different mixtures for 24 h. After the treatment, 100 J2s were added to each well of a 12-well plate and cultured with *H. minnesotensis* strain 3608 at 20 °C for 24 h. The nematodes were washed off using sterilized water. An inverted microscope was used to determine the percentage of nematodes with attached conidia or colonized by the fungi (Xiang et al. 2007). Three replicate wells were performed for each treatment. Statistical differences between each two treatments were determined using the unpaired two-tailed Student’s *t*-test. A *P* value of less than 0.05 was considered to be significant.

## Results

### General Features

Whole genome shotgun sequencing of *H. minnesotensis* strain 3608 was generated using an Illumina Hiseq 2000 sequencer with 128.3-fold coverage and assembled using SOAPdenovo (Li et al. 2009) into 967 scaffolds (N50: 382.4 kb), with an estimated total size of 51.4 Mb (table 1). The genome size is at least 25% larger than those of nematode-trapping fungi in the Orbiliales (Yang et al. 2011) and insect pathogens (Gao et al. 2011; Xiao et al. 2012), but much less than that of *O. sinensis* (Hu et al. 2013) in the Hypocreales.

**Table 1:** Main Features of *Hirsutella minnesotensis* Genome

Features	*H. minnesotensis*
Size (Mb)	51.4
Coverage (fold)	128
Number of scaffolds	967
Scaffold N50 (kb)	382.4
G+C content (%)	52.1
Simple repeat rate (%)	1.33
TEs (%)	34.67
Protein-coding genes	12,702
Gene density (genes per Mb)	247.1
Exons per gene	2.5
Small secreted proteins	494
Unique proteins	1,772
tRNA genes	145

A total of 12,902 protein-coding genes were predicted using a combination of ab initio methods and transcript alignments. The coding regions from the predicted genes constitute 40.1% of the genome with an average gene density of 247 genes per Mb (table 1), which is fewer than most of other nematode-trapping fungi and insect pathogens (Gao et al. 2011; Yang et al. 2011; Xiao et al. 2012; Bushley et al. 2013; Meerupati et al. 2013; Liu et al. 2014). The average 1,624-bp gene length includes an average 1,494 of coding region and 130 bp of noncoding region. The GC content of the genome is 52.1%. A total of 145 transfer RNA (tRNA) genes were predicted from the assembly.

### Expansion of Transposons in *H. minnesotensis* Genome

The richness of TEs is the most remarkable genome feature of this nematode endoparasitic fungus. The massive proliferation of TEs accounts for nearly 32% of the genome (supplementary fig. S1, Supplementary Material online), which is the result of the expansion of certain families. Overall 17,154 occurrences of TEs belonging to two classes were identified. Retrotransposons are dominated by Gypsy (7.18% of the genome) and Copia (4.60%) superfamilies of the long terminal repeat retrotransposons and the I superfamily (4.50%) of the long interspersed elements (supplementary fig. S1, Supplementary Material online). The terminal inverted repeats are the dominant superfamily belonging to Class II DNA transposons (8.10% of the genome).

Like other fungal genomes enriched in repeat sequences (Haas et al. 2009), TEs in *H. minnesotensis* are not uniformly spread across the genome, but are clustered in gene-poor or gene-lacking regions (supplementary fig. S2, Supplementary Material online). The abundant retrotransposons are enriched in the regions in which the gene order is not conserved (supplementary fig. S2, Supplementary Material online), contributing appreciably to the relative expansion of gene-poor regions. In contrast, DNA transposons are relatively evenly distributed across the genomes (supplementary fig. S2, Supplementary Material online). To determine whether the proliferation of TEs in *H. minnesotensis* genome was related to the expansion of specific gene families, GO and Pfam domain enrichment tests were performed for 50-kb genomic windows containing more than 60% TEs. Significant enrichment of genes involved in secondary metabolism and several families of transcription factors was observed in TE-rich regions (supplementary tables S2 and S3, Supplementary Material online). Additionally, large numbers of species-specific genes are also overrepresented in the TE-rich region (supplementary table S4, Supplementary Material online).

### RIP Mutation

RIP mutation is a genome defense mechanism in fungi during which duplicated sequences are mutated from CpA to TpA (Galagan and Selker 2004). A significant RIP was observed in *H. minnesotensis* genome by the high value of TpA/ApT (1.10) and low ratio of (CpA+TpG)/(ApC+GpT) (0.99) (supplementary fig. S3, Supplementary Material online). One homolog (HIM_04934) of DNA methyltransferase RID gene in *Neurospora crassa* (NCU02034) required for RIP was identified in *H. minnesotensis* genome. This supports the active RIP defense mechanism in *H. minnesotensis*.

TEs are frequently a target of RIP mutations, which also preferentially occur in the flanking regions harboring the TEs. Similarly to plant pathogens such as *Fusarium* spp. (Rep and Kistler 2010) and *Dothideomycetes* fungi (Ohm et al. 2012), based on the TpA/ApT index, genes more closely located to TEs are more likely to have a RIP signature in *H. minnesotensis* (supplementary fig. S4, Supplementary Material online). Protein-coding genes involved in secondary metabolism, transcription factors, and members of expanded species-specific multigene families that are overrepresented in the regions flanking TEs have undergone more RIP than other genes.

### Orthologous Gene Groups Specific to Nematode Endoparasitic Fungus and Phylogenomic Relationships

**Fig. 2.—:**
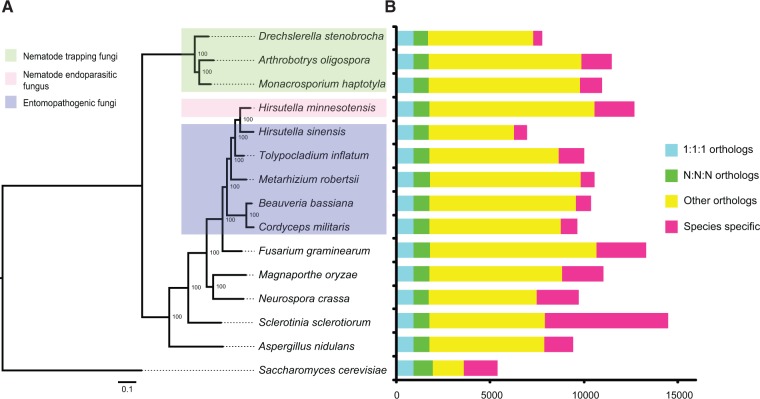
Phylogeny and genome features of *Hirsutella minnesotensis* and other 14 sequenced Ascomycota genomes. (*A*) Genome-based phylogenetic tree of 15 Ascomycota species computed using 898 single-copy orthologs. Bootstrap values are indicated beside the nodes. Life strategies and host preference (green box for nematode-trapping fungi, pink box for nematode endoparasitic fungus, and blue box for insect fungi) are indicated. (*B*) Number of predicted genes and gene conservation among 15 Ascomycota species.

Compared with 14 other sequenced Ascomycota genomes, most orthologous genes (10,570) in *H. minnesotensis* are shared, whereas 2,132 genes are completely unique for this fungus (fig. 2). Despite their conservative relationships, 66.9% of the species-specific genes in *H. minnesotensis* do not contain recognizable Pfam domains (supplementary table S5, Supplementary Material online), indicating their unique gene functions. Of the remaining with Pfam domains, the major functional categories included TEs, secondary metabolism, and transcription factors (supplementary table S5, Supplementary Material online), which were also identified as significantly enriched domains in the fungus (Fisher’s exact test, *P* < 0.05).

A phylogenomic tree generated based on 898 single-copy orthologs revealed that the nematode endoparasitic fungus *H. minnesotensis* clusters with insect pathogens and is closely related to the caterpillar fungus *O. sinensis* (fig. 2). *Hirsutella minnesotensis* diverged from *O. sinensis* around 23.9–33.9 Ma and from *T. inflatum* around 29.7–39.7 Ma (supplementary fig. S5, Supplementary Material online).

### Lectins

The adhesion of nematophagous fungi to their host might be mediated by the interaction between lectins on the surface of trapping devices or adhesive spores and carbohydrate ligands on the nematode cuticle (Nordbringhertz and Mattiasson 1979). Lectins are carbohydrate-binding proteins which can be highly specific to sugar molecules (Sharon and Lis 1989). Protein-coding genes containing lectin domain are identified in nematophagous fungi and insect pathogens. Totally, nematode endoparasitic fungus *H. minnesotensis* has fewer (40) lectin genes than trapping fungi (average 69) (supplementary table S6, Supplementary Material online). Concanavalin A-like lectin binding to α-d-glucose and α-d-mannose is the most abundant family in nematophagous fungi genomes. Compared with trapping fungi, several families including H-type lectin and fucose-specific lectin which bind to *N*-acetyl-d-galactosamine and fucose, respectively, are absent in *H. minnesotensis* genome. In addition, trapping fungi encode a bulb-type lectin which may be used as a defense against nematodes rather than to adhere to them (Liu et al. 2014). Therefore, nematode endoparasitic fungus and trapping fungi have evolved different mechanism to adhere to their host.

Following the sugar inhibition experiment procedure (Nordbringhertz and Mattiasson 1979), we pretreated the nematodes with different lectins and their mixtures. The adhesion rate of *H. minnesotensis* on lectin-treated nematodes significantly decreased compared with the control without lectin treatment (*P* < 0.05), which supports the hypothesis that lectins might involve in the process of recognition and adhesion to the host (supplementary fig. S6, Supplementary Material online).

### Proteases

The nematode cuticle as the primary barrier (Cox et al. 1981) encountered by nematode parasites such as *H. minnesotensis* is rich of protein, and proteases such as serine proteases have been demonstrated to be involved in the infection of nematodes by fungi (Wang et al. 2007, 2009). Total of 293 putative genes encoding proteases, including different exopeptidases and endopeptidases, were identified in *H. minnesotensis*, showing no significant differences with those of nematode-trapping fungi (216–322), but representing fewer genes than those found in three insect pathogens *Me. robertsii* (401), *B. bassiana* (359), and *C. militaris* (341) (supplementary table S7, Supplementary Material online). Approximately 18% of these proteases identified in *H. minnesotensis* contain a signal peptide, which is more likely to be involved in pathogen–host interactions (Ohm et al. 2012), such as their host preference to cyst nematodes (Xiang et al. 2007), whereas entomopathogenic fungi encode more secreted proteases (more than 20%) (supplementary tables S7 and S8, Supplementary Material online) to assist their adaptation on more complex insect cuticles compared with nematode cuticles.

**Fig. 3.—:**
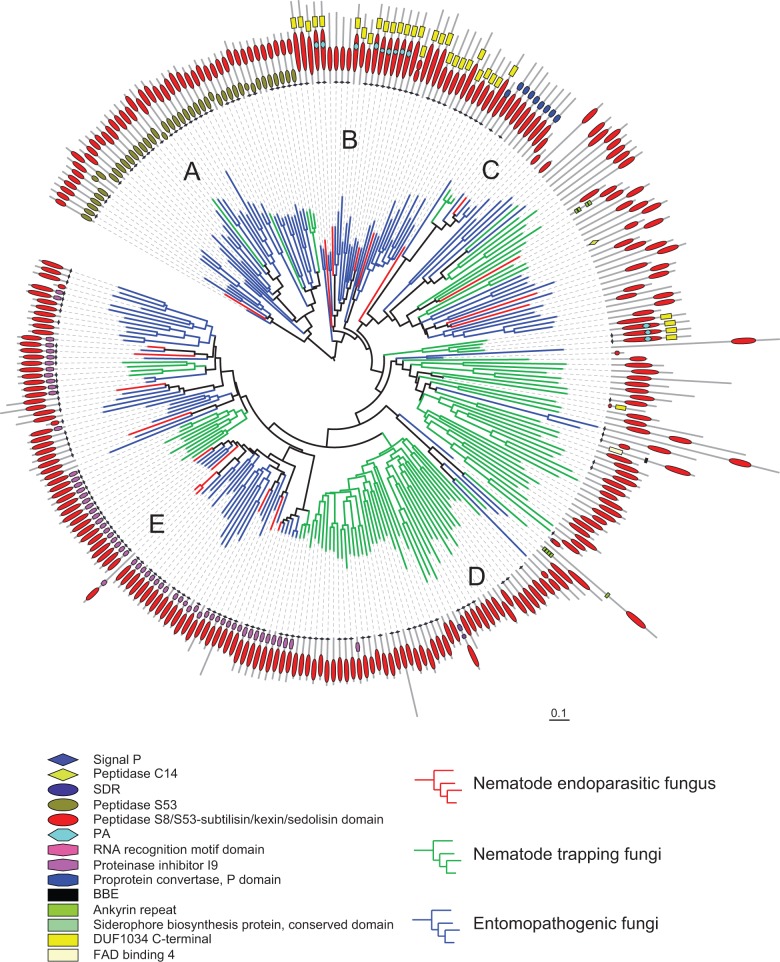
Phylogenetic tree of nematophagous and insect fungal proteinases containing peptidase S8/S53-subtilisin/kexin/sedolisin domains annotated by Pfam. Proteinase genes from one nematode endoparasitic fungus, three nematode-trapping fungi, and five insect fungi are labeled in branch with red, green, and blue color, respectively. Phylogeny was estimated using RAxML (see Materials and Methods). Five clades were identified according to the different domain architecture of proteinase genes. Genes in Clade A contain peptidase S53 domains in combination with the peptidase S8/S53-subtilisin/kexin/sedolisin domains. This combination is found mainly in insect fungi. Most of proteinase genes in nematode endoparasitic fungus are located in Clades B–E. They have more close relationship with those of insect fungi than with trapping fungi. Clades B and C have longer peptidase S8/S53-subtilisin/kexin/sedolisin domains compared with other clades and contain DUF1034 C-terminal and proprotein convertase P domains closest to the conserved domain, respectively. Specially nine genes in Clade B contain PA signature (protease-associated domain) embedded within the peptidase S8/S53 domain. Clade D may have arisen differently; as the majority of proteins belong to nematode-trapping fungi do not have any other domains in combination with the peptidase S8/S53 domain. Half of the genes in Clade D do not carry a signal peptide, thus they do not seem to function extracellularly. Clade E is an expanded group of genes that contain signal peptide and proteinase inhibitor I9 domain on the N-terminal of proteins.

The most enriched secreted protease family in *H. minnesotensis* and other reference fungi are the serine proteases, which comprise approximately 50% of all secreted proteases (supplementary table S8, Supplementary Material online). Serine proteases, especially the subtilases (affiliated into two families: The subtilisin-like protease S8 and the serine-carboxyl protease S53), are involved in lethal activity and the infection process of nematodes (Yang et al. 2005; Wang et al. 2007, 2009). They also assist in the infection processes of entomopathogenic fungi by degrading host cuticles, providing nutrition and disabling antimicrobial peptides (Bagga et al. 2004; Gao et al. 2011). Fewer secreted subtilase coding genes were identified in *H. minnesotensis* compared with the insect pathogen *Me. robertsii* and trapping fungi *A. oligospora* and *Mo. haptotylum*. A phylogenetic tree based on the conserved enzymatic S8/S53-subtilisin/kexin/sedolisin domains (PF00082) revealed that subtilase genes in the endoparasitic fungus diverged from those of insect fungi, supporting the origin of endoparasitic species from insect fungi and their similar functions in protein degradation. Compared with insect fungi, S53 serine proteases, which adapt to the specific insect hosts coupled with S8 proteases (Muszewska et al. 2011), have been lost from nematode-trapping and endoparasitic fungi (fig. 3, Clade A). Although they both belong to the cuticle degrading proteases, the subtilases from nematode-trapping and endoparasitic fungus are not grouped together. Most of the subtilases from trapping fungi only contain the enzymatic domain, whereas subtilases from the endoparasitic fungus contain other functional domains (fig. 3, Clades B–E), such as an N-terminal subtilisin propeptide (proteinase inhibitor I9, PF05922), the cleavage of which activates the enzyme; or the DUF1034 domain (Domain of Unknown Function 1034, PF06280) embedded with proteinase associated (PA, PF02225) domain, which may be involved in recognition of the protein by vacuolar sorting mechanisms (Luo and Hofmann 2001). Therefore, subtilases of nematode endoparasitic fungus and trapping fungi have evolved different functional domain architectures.

### Carbohydrate Active Enzymes

In plant pathogens, carbohydrate-active enzymes are required to degrade complex plant cell wall composed of cellulose, hemicelluloses, and/or pectin (O’Connell et al. 2012). *Hirsutella minnesotensis* has fewer glycoside hydrolases (155), which resembles insect pathogens (average 155) but is significantly less than plant pathogens (average 260, *P* = 0.0203) (supplementary table S9, Supplementary Material online). Similar to insect pathogens, several cellulase families including GH6, GH7, GH12, GH45, and GH61 are contracted or even absent in the *H. minnesotensis* genome. *Hirsutella minnesotensis* as well as insect pathogens also lack several hemicellulase families like GH10, GH11, GH26, GH29, and GH67. With fewer cellulases and hemicellulases, carbohydrate-binding module 1 protein domains enhancing the localization of CAZy enzymes on the surface of crystalline cellulose (Liu et al. 2014) are absent in *H. minnesotensis* (supplementary table S10, Supplementary Material online) genome. In comparison with plant pathogens, *H. minnesotensis* has much fewer putative cutinases (2 vs. average 10) and pectin lyases (2 vs. average 10) (table 2) that are known to be virulence factors for plant pathogens (Xiao et al. 2012). Chitinases are involved in cuticle degradation during insect and nematode infection (Xiao et al. 2012; Liu et al. 2014). Similar to insect pathogens, *H. minnesotensis* encodes more GH18 chitinases (23) involved in cuticle degradation than nematode-trapping fungi (average 11) and plant pathogens (16) (supplementary tables S9 and S11, Supplementary Material online).

**Table 2:** Selected Protein Families Involved in Fungal Pathogenesis in *Hirsutella minnesotensis* and other Fungi

Protein family	HIM	AO	MH	DRE	MRO	BBA	CCM	TIN	OCS	FG	MO	SS	NC	AN	SC
Fungal-specific transcription factors	94	67	67	54	137	141	128	116	44	193	82	69	75	189	30
Zinc finger transcription factors	202	126	38	90	35	132	39	40	35	123	118	55	118	42	83
Pth11-like GPCRs	70	56	34	60	66	42	37	41	32	101	96	45	47	49	10
Protein kinases	139	118	124	104	136	143	117	120	99	126	109	108	106	106	163
Major facilitator superfamily	176	115	109	83	243	221	235	202	81	340	209	177	127	294	62
ABC transporters	56	33	31	32	56	81	68	63	36	61	49	52	36	51	27
Cytochrome P450	135	34	40	20	110	72	53	61	52	104	121	87	41	97	5
Secondary metabolite backbone genes	101	19	17	10	70	48	37	40	28	37	32	29	15	58	1
Serine proteases	99	89	117	56	162	135	114	82	47	119	103	65	51	69	26
Trypsins	4	1	0	1	32	17	8	0	0	2	3	2	1	2	0
Subtilisins	21	43	51	18	47	27	19	16	12	27	26	4	8	3	4
Aspartic proteases	25	32	33	20	33	27	28	20	14	20	21	13	19	11	10
Lipase	60	51	79	14	47	34	36	73	16	57	47	39	20	43	5
Esterase/thioesterase	9	11	10	3	8	4	4	8	0	15	12	8	5	12	0
Glycoside hydrolase	155	226	251	147	193	164	180	147	89	273	278	230	196	268	47
Chitinase	23	15	9	8	27	21	22	16	13	18	15	14	12	17	2
Cutinase	2	3	10	2	2	5	3	2	2	11	13	6	2	3	0
Pectin lyase	2	15	25	3	5	5	3	2	2	21	5	5	4	20	0
Pathogen–host interaction proteins	1,176	760	810	580	1,100	944	874	878	578	1,306	1,025	934	718	1,131	423

Note.—HIM, *Hirsutella minnesotensis*; AO, *Arthrobotrys oligospora*; DRE, *Drechslerella stenobrocha*; MH, *Monacrosporium haptotylum*; OCS, *Ophiocordyceps sinensis*; TIN, *Tolypocladium inflatum*; MRO, *Metarhizium robertsii;* BBA*, Beauveria bassiana;* CCM*, Cordyceps militaris;* FG*, Fusarium graminearum;* MO*, Magnaporthe oryzae;* SS*, Sclerotinia sclerotiorum;* NC, *Neurospora crassa;* AN, *Aspergillus nidulans;* SC*, Sacchromyces cerevisiae.*

### Signal Transduction

Fungal GPCRs are key components of signaling pathways for pheromone/nutrient sensing and host recognition (Xue et al. 2008; Gao et al. 2011). Most GPCRs encoded by *H. minnesotensis* resemble the rice-blast fungus *Magnaporthe oryzae* Pth11-like proteins which are required for sensing the host surface stimuli to produce appressorium in plant pathogens (DeZwaan et al. 1999). Similar to nematode-trapping fungi *A. oligospora* and *Mo. haptotylum* and the insect pathogen *Me. robertsii* with wide host range, *H. minnesotensis* has more Pth11-like receptors (42) than most insect pathogens (average 22) (supplementary table S12, Supplementary Material online). Besides, one homolog of *Saccharomyces cerevisiae* GPR-1, which is activated in yeast during nitrogen starvation (Xue et al. 1998), is also predicted in *H. minnesotensis* and nematode-trapping fungi, whereas most insect pathogens (except *Me. robertsii*) lack this GPR1-like GPCR (supplementary table S12, Supplementary Material online).

Reversible protein phosphorylation by protein kinase plays critical roles in the regulation of fungal growth, pathogenesis, stress responses, toxin production, and sexual reproduction (Wang et al. 2011). The nematode endoparasitic fungus *H. minnesotensis* encodes more protein kinases (139) than the trapping fungi (average 115) (supplementary table S13, Supplementary Material online). Especially, serine/arginine-rich protein kinases involved in the modulation of salt tolerance, membrane potential, and potassium transporter in *S. cerevisiae* (Forment et al. 2002) are expanded in *H. minnesotensis* genomes (6) compared with those of trapping fungi (average 1) (supplementary table S13, Supplementary Material online).

Physiological responses following the signal transduction are regulated by activation of different transcription factors. *Hirsutella minnesotensis* encodes large number of transcription factors (979) more than nematode-trapping fungi (average 374) and insect pathogens (average 422) (supplementary table S14, Supplementary Material online). The most of *H. minnesotensis* gene expansion involves in Zn2/Cys6, centromere protein B, homeodomain-like, CCHC zinc finger, and bZIP transcription factors. These families especially the Zn2/Cys6 type play important role in regulating protein and polysaccharide degradation (Pel et al. 2007). Overall, compared with nematode-trapping fungi and insect pathogens, *H. minnesotensis* has evolved different signal controls for response to the environment stimuli.

### Expansion and Diversity of Secondary Metabolic Genes

Genes encoding secondary metabolic enzymes such as PKS, the NRPS, and the TPS are significantly expanded in *H. minnesotensis* genome (supplementary table S15, Supplementary Material online, *P* < 0.05). Totally, 101 core genes were identified, compared with 10–19 in trapping fungi and 28–70 in insect fungi (supplementary table S15, Supplementary Material online). The numbers of Type I PKS biosynthetic genes possessed by *H. minnesotensis* (26) are significantly more than trapping fungi (average 2, *P = *0.0151) and insect pathogens (average 13, *P = *0.0014). *Hirsutella minnesotensis* also encodes abundant of putative NRPS genes (23) in comparison with trapping fungi (average 2) and insect pathogens (average 12). Phylogenetic analyses indicate the PKS genes of nematode endoparasitic fungus evolved from those of insect pathogens (supplementary fig. S7, Supplementary Material online). Three, six, five, and ten PKS genes are clustered with other genes involved in the biosynthesis of pigments, mycotoxins, lovastatin, and alternapyrone, respectively (supplementary fig. S7, Supplementary Material online), indicating that *H. minnesotensis* might produce secondary metabolites with similar structures. Six NRPS and two terpene genes are unique to the nematode endoparasitic fungus, suggesting lineage-specific expansion of these families in *H. minnesotensis* genome.

**Fig. 4.—:**
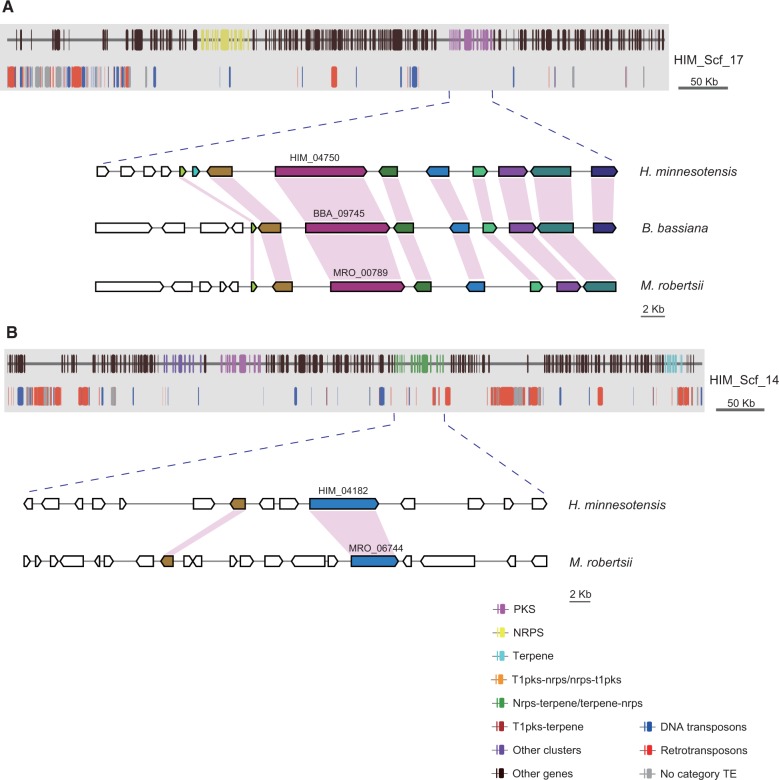
Genomic landscape of selected scaffolds containing secondary metabolite gene clusters in *Hirsutella minnesotensis*. (*A*) Gene clusters located in TE-poor regions are syntenic between *H. minnesotensis* and other insect fungi. (*B*) Gene clusters located in TE-rich regions are expanded and diverse without synteny.

The core genes for secondary metabolite production in fungal genomes are typically located in clusters with other accessory genes, such as those encoding dehydrogenases, methyltransferases, acetyl transferases, prenyltransferases, oxidoreductases, and cytochrome P450s (Gao et al. 2011). In total, 94 secondary metabolite gene clusters were identified in *H. minnesotensis* genome. Although most PKS and NRPS genes are orthologous across the nematophagous fungi and insect fungi, the accessory genes in the secondary metabolite gene clusters are more dynamic. Only five secondary metabolism gene clusters are shared between *H. minnesotensis* and other insect pathogens with limited synteny (fig. 4*A*). This cluster diversity seems to have resulted from gene duplication or loss related to TEs associated with the secondary metabolism gene clusters (fig. 4*B*). Thirty-two of 94 gene clusters in *H. minnesotensis* are located in TE-rich regions (>60% TE).

### *Transcriptomics Analysis during Infection of* H. minnesotensis

*Hirsutella minnesotensis* was investigated about the infection process on nematodes by the RNA-seq (RNA sequencing) approach. The relative expression of all *H. minnesotensis* genes was determined by Illumina sequencing across four infection stages, including i) *H. minnesotensis* cultures on CMA medium free of nematodes to produce large amount of spores (0 h), ii) spore adhesive stage after 8 h of culture with nematode challenge (8 h), iii) infection peg formation stage after 16 h of nematode challenge (16 h), and iv) stage of fungal mycelium growth in the body of nematode after 36 h (36 h). In total, 9,277 of 12,702 predicted genes were expressed during the infection process, representing 73.0% of the *H. minnesotensis* transcriptome. After infection on nematodes for 8, 16 and 36 h, 1,433, 1,448 and 973 putative protein-coding genes were significantly up- or downregulated (*P* < 0.05), respectively, compared with that of the culture on CMA without nematodes. Many of these differentially expressed genes encode known proteins (approximately 70%), a significantly greater proportion than for nondifferentially expressed genes.

**Fig. 5.—:**
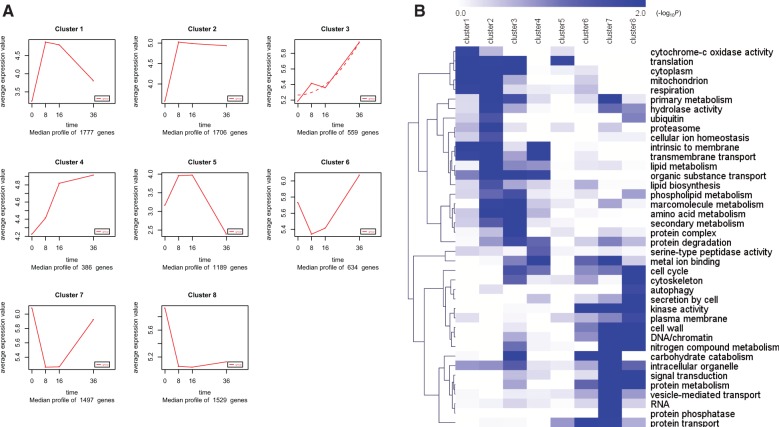
Dynamic progression of *Hirsutella minnesotensis* transcriptome at different parasitism stages on *Caenorhabditis elegans*. (*A*) K-means clustering showing the expression profile of the *H. minnesotensis* transcriptome. Eight clusters were identified and presented along the four parasitism stages (0, 8, 16, and 36 h postinoculation) from 9,277 differentially expressed genes. (*B*) Functional category enrichment (heat map) among the eight clusters. Blue, significant enrichment; white, nonsignificant.

GO terms and Pfam domains were used to assign genes to functional categories and group the genes by parasitic dynamics using the K-Means clustering algorithm. Eight main clusters (fig. 5*A*, clusters 1–8) accounted for about 93% of the differentially expressed genes in the four stages. Most of the blue bins showed enrichment for particular clusters of gene expression (fig. 5*B*). Genes that encode proteins involved in translation, cytoplasm, respiration activity, hydrolase activity, proteasome, transmembrane transport, lipid metabolism, and organic substance transport are greatly enriched in clusters 1 and 2, representing genes that are expressed at the highest levels at the spore adhesion stage. High expression of genes responsible for the proteasome and hydrolase activity indicated their important role in cuticle degradation. Genes that showed peak expression during the period when fungal mycelium growth within the nematode body included those that are required for phospholipid metabolism, macromolecular metabolism, amino acid metabolism, protein degradation, serine-type peptidase activity, and secondary metabolism, suggesting that the fungal transcriptome undergoes detoxification in host body, degrades nematode protein substrates, and involves nutrient uptake.

Recognition and adhesion are the first two steps of fungal invasion of nematodes. In the genome of *H. minnesotensis*, two concanavalin A-like lectins of 18 secreted lectin proteins were upregulated at the adhesive stages (supplementary table S16, Supplementary Material online). In addition, *H. minnesotensis* encodes 16 adhesive proteins containing a CFEM (eight cysteine-containing extracellular membrane domain, PF05730) that are used by pathogenic fungi to recognize and attach to their hosts (Kulkarni et al. 2003), and four of them (HIM_02815, HIM_03774, HIM_04712, and HIM_08135) were significantly upregulated (log2-fold change > 2, *P* < 0.05) at the adhesive stage (8 h) (supplementary table S16, Supplementary Material online).

Secreted enzymes, including one pepsin-like aspartic peptidases (HIM_11269) and five subtilisin serine peptidases (HIM_04064, HIM_04124, HIM_04145, HIM_05938, and HIM_09336), were significantly upregulated (log2-fold change > 2, *P* < 0.05) (supplementary fig. S8, Supplementary Material online) during the penetration stage and may be involved in degrading the nematode cuticle. One gene involved in cell wall assembly (HIM_08835; PF09346), one in cell wall beta-glucan synthesis (HIM_02479; PF10342), and three genes containing cell wall galactomannoprotein (HIM_02846, HIM_00809; PF12296; HIM_11494) were highly expressed during the infection peg formation. Swelling at the peg tip indicates the stress response to reactive oxygen, nitrogen, and chlorine species generated by the defense action of the host. Four genes (HIM_00696, HIM_03743, HIM_08072, and HIM_08834) encoding thioredoxin (PF00085) acting as antioxidants were activated during infection stage.

After the interaction of the fungus and the nematode during the peg stage, the fungus conquers the nematode defense and consumes the nematode body content for growth. Both the endopeptidases M4 and M43, and exopeptidases M14 and M28 (zinc-metallopeptidases) were highly expressed at this stage (36 h). In addition, one general substrate transporter (HIM_03796), two oligopeptide transporters (HIM_04215, HIM_06101), and one amino acid transporter (HIM_07163) were also strongly activated to facilitate the nutrient transportation. The transcriptome profile of *H. minnesotensis* also indicated that the enriched genes for secondary metabolites were upregulated: 17, 6, and 2 different clusters were induced before penetration, during penetration, and at the mycelium growth stage, respectively (supplementary fig. S9 and table S16, Supplementary Material online).

Like the infection structures of appressoria for entomopathogenic fungi (Gao et al. 2011) and the constricting ring for nematode-trapping fungus (Liu et al. 2014), cell remodeling by the protein kinase C (PKC) signal transduction pathway is important for peg formation in *H. minnesotensis*. The PKC1 (HIM_00683), Bck1 (HIM_03632, MAPKKK), and Mkk1 (HIM_07373, MAPKK) genes were relatively highly expressed at 16 h compared with 8 h (supplementary table S17, Supplementary Material online). Adaptation to stress (osmotic, oxidative, acid, and heat) mainly occurs through the high osmolarity glycerol (HOG) pathway (Román et al. 2007). The MAPK Hog1 (HIM_00488) gene was also relatively highly activated at the peg formation stage (supplementary table S17, Supplementary Material online). The significant upregulation of protein kinase A (PKA) during the last stage revealed the importance of the cyclic AMP-protein kinase A pathway for filamentation (supplementary table S17, Supplementary Material online). Meanwhile, two genes (HIM_01527 and HIM_07761) resembling the STM1-like GPCR responsible for triggering adaptation to nitrogen starvation were upregulated during the whole infection process. Overall, the nematode endoparasitic fungus is able to respond to diverse host-derived stimuli using distinct or shared signal pathways involving mitogen-activated protein kinase and cAMP-PKA to carry out their infection cycle on the nematode hosts (supplementary fig. S10, Supplementary Material online).

## Discussion

*Hirsutella minnesotensis* is one of the most important endoparasites of SCNs. Different from other animal pathogens such as nematode-trapping fungi and entomopathogenic fungi, *H. minnesotensis* produces adhesive spores to adhere to, penetrate, and eventually kill their nematode host. The analysis of *H. minnesotensis* genome compared with genomes of other insect pathogens in Hypocreales and trapping fungi in Orbiliales and the transcriptome of *H. minnesotensis* on *C. elegans* have provided insights into the evolution and life strategy of nematode endoparasites.

The most significant characteristic of *H. minnesotensis* genome compared with other animal pathogen genomes (except *O. sinensis*) is that *H. minnesotensis* contains a strikingly rich and diverse population of TEs, associated with its relatively larger genome size. An expanded TE-rich genome is also a feature of some biotrophic obligate plant pathogens, such as *Blumeria graminis* (Spanu et al. 2010) and *Puccinia graminis* (Duplessis et al. 2011), the ectomycorrhizal fungus *Tuber melanosporum* (Martin et al. 2010), and the caterpillar fungus *O. sinensis* (Hu et al. 2013) that are closely associated with their hosts. The disruption of gene collinearity in the TE-rich regions (fig. 4) increased recombination to mediate chromosomal rearrangements, deletions, and duplications, as well as greater sequence diversity (Raffaele and Kamoun 2012). A positive correlation between recombination rates and the distribution of TEs in clusters that break synteny between *Ma. oryzae* and other fungi has also been noted, indicating increased rates of structural variation in TE-rich regions (Thon et al. 2006). Overrepresentation of secondary metabolism genes, zinc finger transcription factors, and species-specific genes in these TE-rich regions of *H. minnesotensis* indicates the proliferation and mobility of TEs contribute to the duplication, expansion, and diversity of these gene families. As in other fungi the TE-rich regions were analyzed to contain genes associated with host adaptations (Spanu et al. 2010; Duplessis et al. 2011; Klosterman et al. 2011), the proliferation of TEs in *H. minnesotensis* might be responsible for the close association of *H. minnesotensis* with its host nematodes and could explain the obligate parasitism of nematode in nature (Sturhan and Schneider 1980; Jaffee and Zehr 1985; Jaffee et al. 1991; Timper and Brodie 1994; Velvis 1995; Chen and Reese 1999). Repetitive sequences in fungal genomes appeared to be the target of RIP mutations, and RIP mutations can leak to regions flanking the repetitive sequences. This characteristic in *H. minnesotensis* genome is consistent with the plant pathogens such as *Fusarium* spp. (Rep and Kistler 2010) and *Dothideomycetes* fungi (Ohm et al. 2012) in which higher mutation rate associated with the RIP process accelerates the evolution of effector genes that locate in repeat-rich regions. (Rouxel et al. 2011). The potential evolutionary benefit of colonization of TEs and genes encoding secondary metabolites and transcription factors in *H. minnesotensis* is a higher rate of mutation caused by RIP, which in turn may lead to a higher rate of evolution. This would allow the fungus to adapt more quickly to the host nematode’s defenses and ecological niches, and thereby provide an advantage in the “arms race” against their hosts (Jiang XZ, Shu C and Liu XZ, unpublished data).

*Hirsutella minnesotensis* is a member of the order Hypocreales which is an important group of fungi that has various lifestyles, including plant pathogens, endophytes, hyperparasites, insect pathogens, and nematode parasites. The phylogenomic analyses demonstrated that *H. minnesotensis* is evolutionary close to the insect fungi *T. inflatum* and *O. sinensis* in the family Ophiocordycipitaceae and evolved into nematode endoparasite independently out of nematode-trapping fungi lineage (fig. 2). This evidence supported the viewpoint that this species has divergent after fungi adapted to insect hosts. This inference is strengthened by the consistent existence of genes for cuticle degrading enzymes and contraction of genes for cellulose/hemicellulose degrading enzymes. As both insects and nematodes are rich of protein and chitin, serine proteases and chitinases involved in cuticle degradation are also abundant in nematode endoparasitic fungus and are most closely related to those genes of insect pathogens. Both *H. minnesotensis* and insect pathogens lost genes involved in cellulose and hemicellulose degradation, indicating that the nematode endoparasitic fungus shares more similarities with insect pathogens than with plant pathogens or saprophytic fungi. Although nematodes are the hosts of both trapping fungi and endoparasitic fungi, trapping fungi are considered to be divergent from saprophytic fungi which is attributed to the relative abundance of enzymes for cellulose degradation (Yang et al. 2012; Liu et al. 2014).

As *H. minnesotensis* and insect pathogens are closely related and evolved independently of nematode-trapping fungi, we assume that the nematode endoparasitic fungus and trapping fungi have evolved different pathogenesis mechanisms. Our comparative genomics analyses found that *H. minnesotensis* contains fewer lectin genes than trapping fungi, especially than the adhesive networks producer *A. oligospora* and the adhesive knobs producer *Mo. haptotylum*. The interaction between lectins on trapping surface and carbohydrate ligands on nematode surface was considered to mediate the adhesion of nematodes to trapping fungi (Nordbringhertz and Mattiasson 1979). This adhesion was also indicated to exist between *H. minnesotensis* spores and nematodes by lectin inhibition experiment in our study. Fungi producing adhesive device types such as *A. oligospora* and *Mo. haptotylum* might need strong adhesion by lectins to prevent their prey nematode from escaping. However, for endoparasitic fungus *H. minnesotensis*, once its spore adheres to the host cuticle, it will fall from the conidiophores, move with the host, and eventually infect the nematode. Therefore, *H. minnesotensis* might not require many lectins for adhesion. *Drechslerella stenobrocha* with constricting-ring mechanical trapping mechanism also encodes fewer lectins (Liu et al. 2014). These differences of adhesion to host are also reflected in the contraction of several cell surface proteins including carbohydrate-binding WSC domain containing proteins and GLEYA proteins in nematode endoparasitic fungus compared with adhesive trapping fungi. Both WSC and GLEYA are carbohydrate-binding domains found in fungal adhesins (Linder and Gustafsson 2008; Andersson et al. 2013).

In *H. minnesotensis*, genes encoding plant cell wall degrading enzymes are reduced in number compared with plant pathogens and nematode-trapping fungi. Compared with *A. oligospora* and *Mo. haptotylum*, for example, *H. minnesotensis* exhibited fewer or no genes encoding enzymes to degrade celluloses, hemicelluloses, cutin or pectin. Coupled with these losses in plant degrading enzymes, there are abundance of protease families in *H. minnesotensis* relative to trapping fungi. Serine proteases especially the subtilase type including subtilisin-like proteinases (S8) and serine-carboxyl proteinases (S53) involve in the penetration and colonization on hosts for nematophagous fungi and entomopathogenic fungi (Li et al. 2010; Muszewska et al. 2011). Consistent with previous studies (Li et al. 2010), the higher homology is found between *H. minnesotensis*-predicted subtilase genes and those of entomopathogenic fungi compared with those of nematode-trapping fungi, which also reflects the differences from trapping fungi in pathogenesis mechanisms. Similar to entomopathogenic fungi, subtilases in *H. minnesotensis* contain the enzymatic domain combined with other functional domains, whereas most subtilases in trapping fungi only contain the core domain. Nematode endoparasitic fungus does not produce trapping devices; therefore, it is likely to rely mainly on extracellular enzymes, including the subtilases (Li et al. 2010). Thus, the variety of subtilases with different functional domains in the endoparasitic fungus may facilitate the penetration and digestion of nematode cuticles. However, serine-carboxyl proteinases containing S53 domains that can couple with S8 proteases (Muszewska et al. 2011) to degrade insect-source proteins are lost in *H. minnesotensis* and trapping fungi, which might limit their degradative ability on insect cuticles. Thus, *H. minnesotensis* adapts to nematodes as host rather than insects by protease gain and loss from its relative entomopathogenic fungi. Moreover, *H. minnesotensis* has greater capacity of cuticle degradation than trapping fungi by encoding more chitinase to degrade chitin, a component of nematode cuticle (Liu et al. 2014). Overall, these changes in plant cell wall degrading enzymes and serine proteases suggest that *H. minnesotensis* is not typical saprophytic species in that it maintains a close association with protein-rich nematodes.

The *H. minnesotensis* genome contains very rich repertories of secondary metabolism genes compared with entomopathogenic and trapping fungi. The gene clusters are very diverse, with only a few clusters in *H. minnesotensis* showing limited synteny with other fungi. As each secondary metabolism gene cluster is probably involved in the biosynthesis of a specific metabolite (Collemare et al. 2008), this species can be expected to produce large and diverse spectra of secondary metabolites, some could be novel bioactive molecules. Secondary metabolism genes in insect pathogens are responsible for the production of certain insect toxins such as destruxin (Wang et al. 2012), beauvericin (Xu et al. 2008), and bassianolide (Xu et al. 2009) and determine host range (Gao et al. 2011). Secondary metabolite genes that are induced at the adhesive stage may synthesize small molecules for signaling and host manipulation, similar to effectors produced by plant pathogenic fungi (O’Connell et al. 2012), whereas those highly expressed at the peg formation and mycelium growth stages may produce toxins that help to counter host defenses and nutrient absorption (Roberts and St Leger 2004). Nematodes are very tiny animals occurring in the complicated soil habitat. After infection, the nematodes are too small to support *H. minnesotensis* to conquer the soil fungistasis and other fungal and bacterial competitors (Garbeva et al. 2011). Several antibiotics are easily detected, extracted, and identified from *H. minnesotensis* cultures (data unpublished), including the wide-spectrum antibiotic phomalactone (5,6-dihydro-5-hydroxy-6-prop-2-enyl-2H-pyran-2-one) actively against entomopathogenic fungi, plant pathogenic fungi, insects and plant (Krasnoff and Gupta 1994; Kim et al. 2001), the insect toxin musacin D (Burkhardt et al. 1996; Schneider et al. 1996), and the phytotoxin musacin E [dihydro-5-(1-hydroxy-2-butenyl)-2(3H)-furanone] (Trisuwan et al. 2009). So we hypothesize that secondary metabolites, particularly the antibiotics, help the fungus survive and colonize in soil by inhibiting microbial competitors. Confirmation of these secondary metabolites and their functions will be addressed in future researches.

The lifestyle of *H. minnesotensis* would require a different signaling machinery to respond to a variety of environmental stimuli. In this light, *H. minnesotensis* as well as trapping fungi such as *A. oligospora* and *Mo. haptotylum* contain much more numbers of Pth11-like G-proteins, which have been demonstrated to involve in physiological processes in the plant pathogen *Ma. oryzae* (DeZwaan et al. 1999), than entomopathogenic fungi (except *Me. robertsii*). *Hirsutella minnesotensis* and trapping fungi also encode GPR1 nutrient sensors, which are absent in entomopathogenic fungi. These similar expansion of GPCRs associated with functions necessary for nutrient sense and host recognition reflects convergent evolution between nematode endoparasitic fungus and trapping fungi. However, *H. minnesotensis* encodes expanded protein kinase involved in regulation of fungal growth, pathogenesis, toxin production, and sexual reproduction compared with trapping fungi especially has a complex signal transduction cascade controlling stress response. Moreover, transcription factors belonging to Zn2/Cys6 fungal type, zinc finger, and bZIP which are expressed during pathogenesis (Larriba et al. 2014) are well represented in the *H. minnesotensis* genome, indicating that *H. minnesotensis* might make different physiological responses regulated by activation of different transcription factors. These analyses highlight the importance of identification of signal and transduction pathways involved in nematode endoparasitism.

The transcriptomes during nematode infection process demonstrate that *H. minnesotensis* finely expresses and regulates genes to finish their infection cycle on their host. At adhesion stage, two upregulated concanavalin A-like lectins were involved in adhesion between nematode and endoparasitic fungus. Several upregulated serine proteases were responsible for protein-rich cuticle degradation during fungal penetration. Subsequently, similar to entomopathogenic fungi (Gao et al. 2011) and trapping fungus *D. stenobrocha* (Liu et al. 2014), cell remodeling by the PKC signal transduction pathway was activated for peg formation. The high osmolarity glycerol (HOG) pathway was induced under the osmotic and oxidative stress from the nematode host body, with upregulation of several thioredoxin. After fungal mycelium growth in the nematode body, exo- and endo-metallopeptidases with potential roles in pathogenicity (Martinez et al. 2012) were significantly expressed, indicating their important role in efficient degradation of nematode protein substrates for nutrients. The cAMP-PKA pathway may be responsible for filamentation in the host body. Thus, comparative genomics and transcriptomics revealed key genes and putative pathways active during nematode endoparasitism and provided new insights into the life strategy of this fungus.

In conclusion, we sequenced the first genome of nematode endoparasitic fungus and compared with those of other animal pathogens. Phylogenomic analysis revealed that *H. minnesotensis* evolved after the divergence of insect pathogens which also supported by the high similarity of serine proteases and contraction of plant cell wall degrading enzymes between *H. minnesotensis* and insect pathogens. Compared with trapping fungi, *H. minnesotensis* genome contains reduced number of cellulose/hemicellulose degrading enzymes and fewer lectins for adhesion, but possesses expanded gene inventories for protein degradation by multiple functional domains-subtilases, signal transduction for nutrient sensing and host recognition and rich gene repertories of secondary metabolism. The upregulation of lectins, serine proteases, and secondary metabolism genes during the infection of nematodes indicated that these genes are involved in adhesion, recognition, cuticle degradation, signaling, and detoxification. The comparative genomics and transcriptomics provide comprehensive understanding of the evolution and life strategy of this species and highlight the future investigation of the molecular mechanism underlying this fascinating life style of nematode endoparasitic fungi.

## Supplementary Material

Supplementary figures S1–S10 and tables S1–S17 are available at *Genome Biology and Evolution* online (http://www.gbe.oxfordjournals.org/).

Supplementary Data
