# Supplementary material for: Comparative Genomics and Transcriptomics Analyses Reveal Divergent Lifestyle Features of Nematode Endoparasitic Fungus Hirsutella minnesotensis
Source: Genome Biol Evol. 2014 Oct 30;6(11):3077–93. doi: 10.1093/gbe/evu241 (PMC4255773; doi:10.1093/gbe/evu241)
Supplement: Supplementary Data [file supp_6_11_3077__index.html]

Comparative genomics and transcriptomics analyses reveal divergent lifestyle features of nematode endoparasitic fungus Hirsutella minnesotensis — Comparative Genomics and Transcriptomics Analyses Reveal Divergent Lifestyle Features of Nematode Endoparasitic Fungus Hirsutella minnesotensis — Supplementary Data 

# Comparative Genomics and Transcriptomics Analyses Reveal Divergent Lifestyle Features of Nematode Endoparasitic Fungus *Hirsutella minnesotensis*

## Supplementary Data

files

**Files in this Data Supplement:**

- Supplementary Data - pdf file
- Supplementary Data - docx file
- Supplementary Data - xlsx file
