# Supplementary material for: Comparative Genomics and Transcriptomics Analyses Reveal Divergent Lifestyle Features of Nematode Endoparasitic Fungus Hirsutella minnesotensis
Source: Genome Biol Evol. 2014 Oct 30;6(11):3077–93. doi: 10.1093/gbe/evu241 (PMC4255773; doi:10.1093/gbe/evu241)
Supplement: Supplementary Data [file supp_evu241_Figure_S.pdf]

**Classification**		**Structure**	***H. minnesotensis***		
**Order**	**Superfamily**		**Number of Consensus Sequences**	**Number of Occurrences**	**% Assembly Coverage**
**Class Ⅰ (retrotransposons)**					
LTR	Copia	GAG AP INT RT RH	70	2,035	4.60%
	Gypsy	GAG AP RT RH INT	93	2,880	7.18%
	Others LTR		15	590	2.22%
LINE	I	ORF1 APE RT RH	47	2,260	4.50%
	R2	RT EN	1	7	0.03%
	Others LINE		3	86	0.14%
SINE			2	15	0.01%
Elements confused			102	2,789	4.93%
**Class Ⅱ (DNA transposons)- Subclass 1**					
TIR	Tc1-Mariner	Tase*	62	1,720	3.03%
	hAT	Tase*	54	2,012	2.57%
	MuDR	Tase*	27	1,048	1.72%
	PiggyBac	Tase*	14	272	0.51%
	P	Tase*	3	41	0.07%
	Others TIR		7	184	0.20%
**Class Ⅱ (DNA transposons)- Subclass 2**					
Helitron	Helitron	RPA Y2 HEL	9	430	1.45%
Elements confused			12	191	0.26%
**Elements not categorized**			**14**	**594**	**1.23%**
**Total**			**535**	**17,154**	**34.67%**

Fig. S1

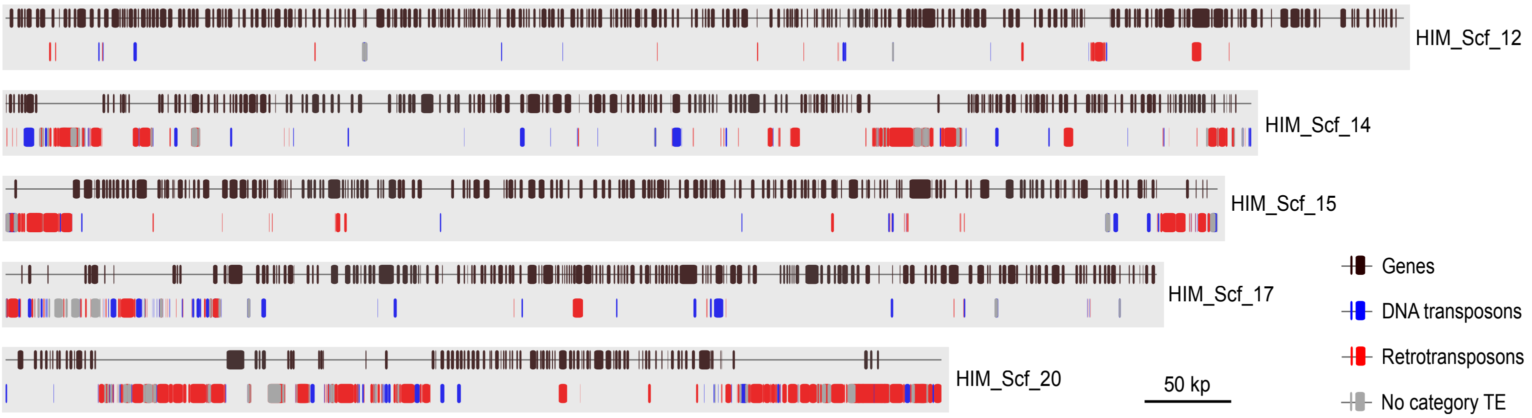
HIM_Scf_12
HIM_Scf_14
HIM_Scf_15
HIM_Scf_17
HIM_Scf_20
50 kp
Genes
DNA transposons
Retrotransposons
No category TE

Fig. S2

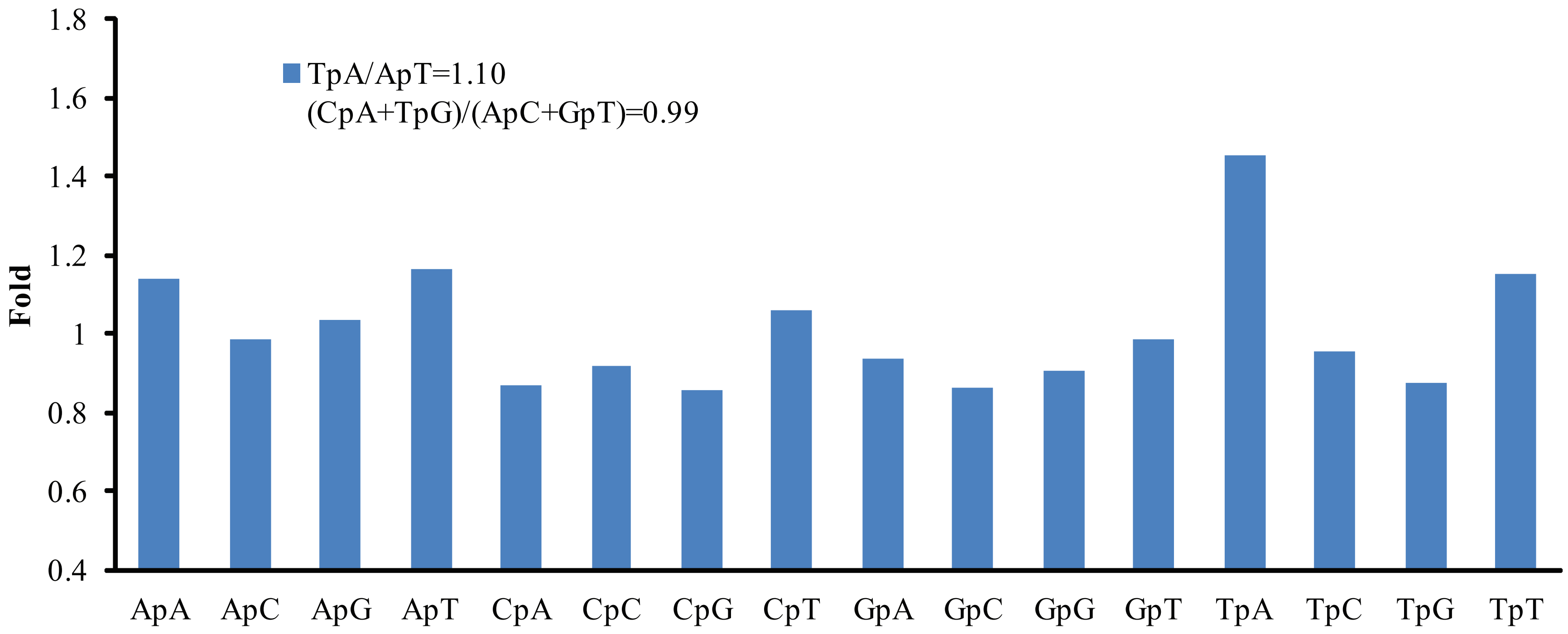
TpA/ApT=1.10
(CpA+TpG)/(ApC+GpT)=0.99
Fold
1.8
1.6
1.4
1.2
1
0.8
0.6
0.4
ApA
ApC
ApG
ApT
CpA
CpC
CpG
CpT
GpA
GpC
GpG
GpT
TpA
TpC
TpG
TpT

Fig. S3

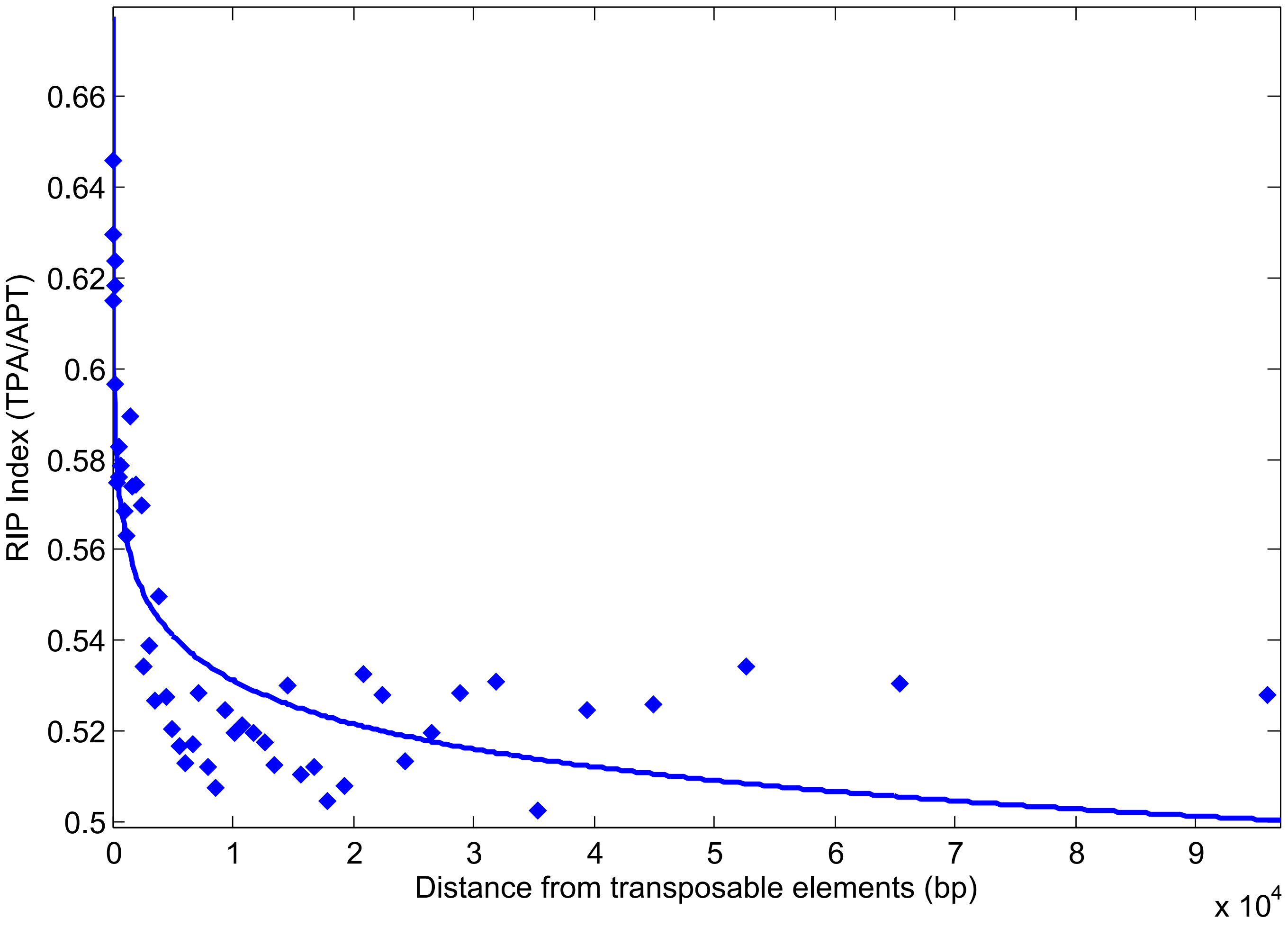
0.66
0.64
0.62
0.6
0.58
0.56
0.54
0.52
0.5
RIP Index (TPA/APT)
0
1
2
3
4
5
6
7
8
9
Distance from transposable elements (bp)
x 10^4

Fig. S4

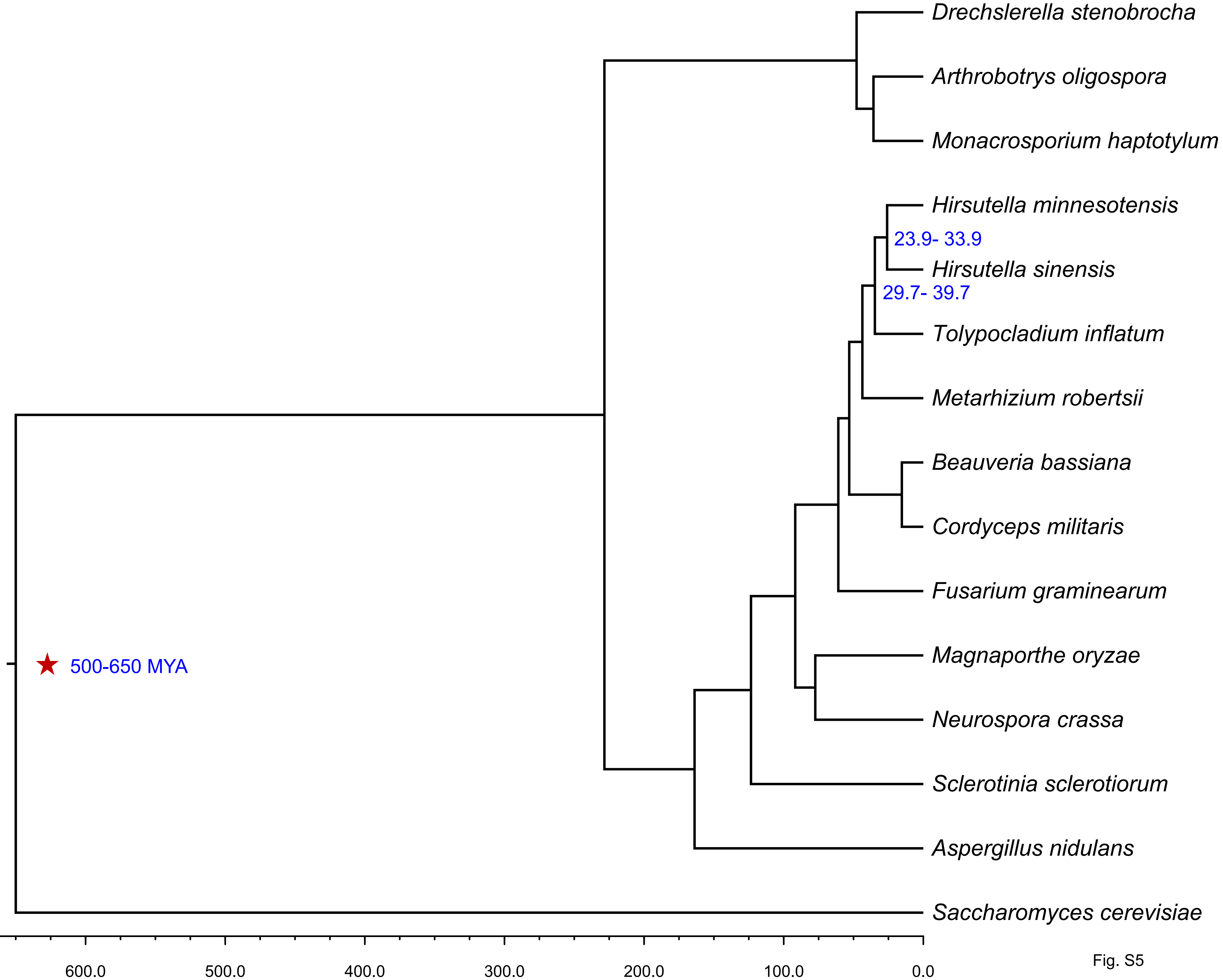
Drechslerella stenobrocha
Arthrobotrys oligospora
Monacrosporium haptotylum
Hirsutella minnesotensis
23.9- 33.9
Hirsutella sinensis
29.7- 39.7
Tolypocladium inflatum
Metarhizium robertsii
Beauveria bassiana
Cordyceps militaris
Fusarium graminearum
Magnaporthe oryzae
Neurospora crassa
Sclerotinia sclerotiorum
Aspergillus nidulans
Saccharomyces cerevisiae
500-650 MYA
600.0
500.0
400.0
300.0
200.0
100.0
0.0

Fig. S5

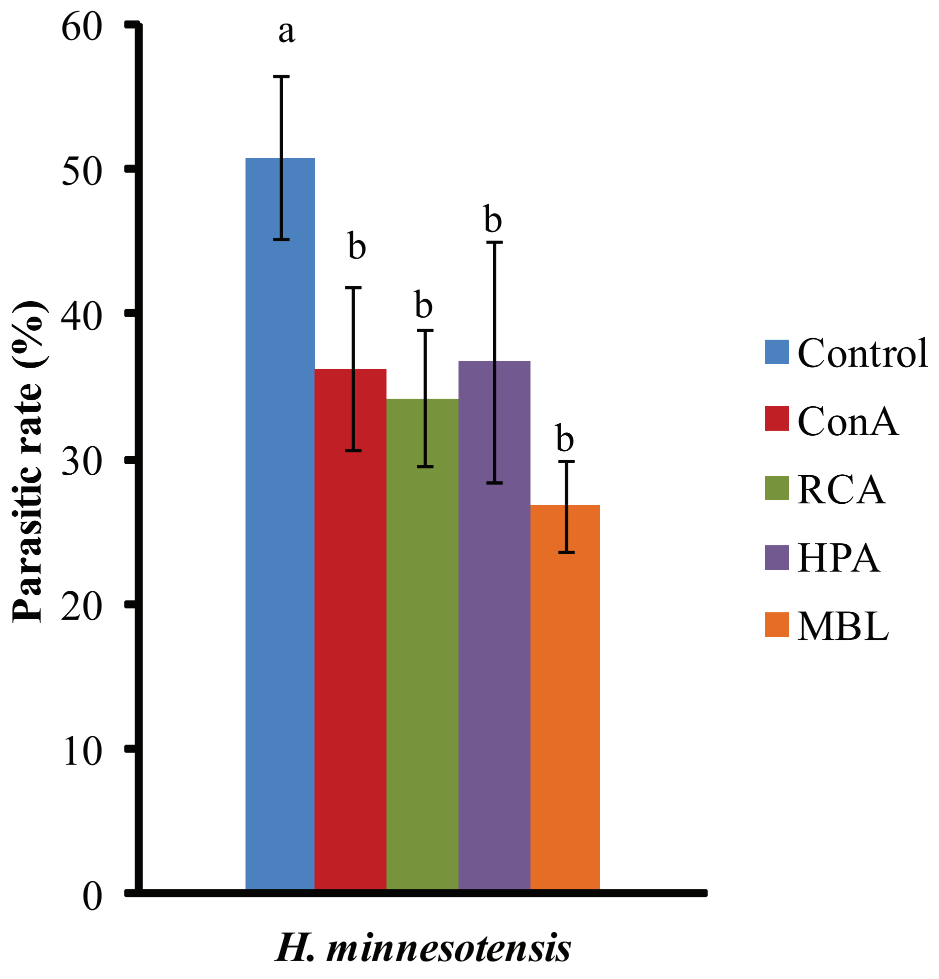

60
50
40
30
20
10
0
Parasitic rate (%)
a
b
b
b
b
Control
ConA
RCA
HPA
MBL
H. minnesotensis

(A)

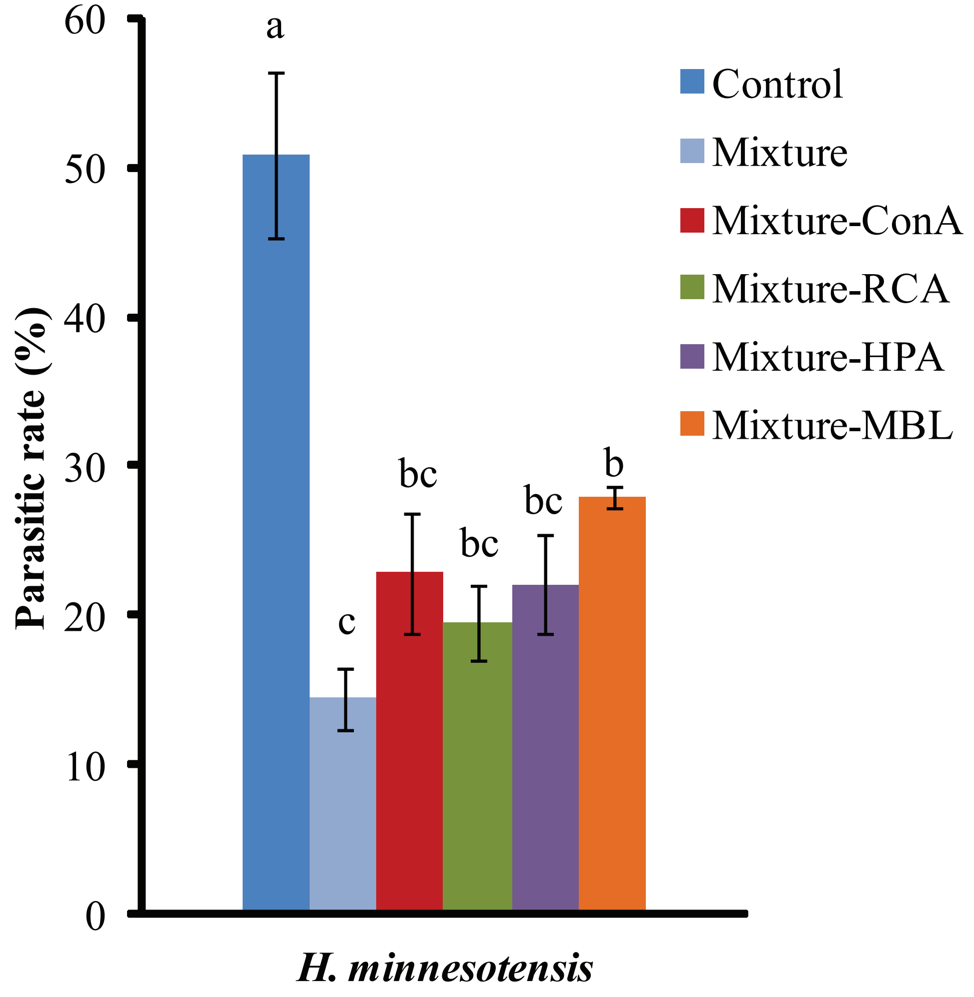

60
50
40
30
20
10
0
Parasitic rate (%)
a
c
bc
bc
bc
b
Control
Mixture
Mixture-ConA
Mixture-RCA
Mixture-HPA
Mixture-MBL
H. minnesotensis

Fig. S6

(B)

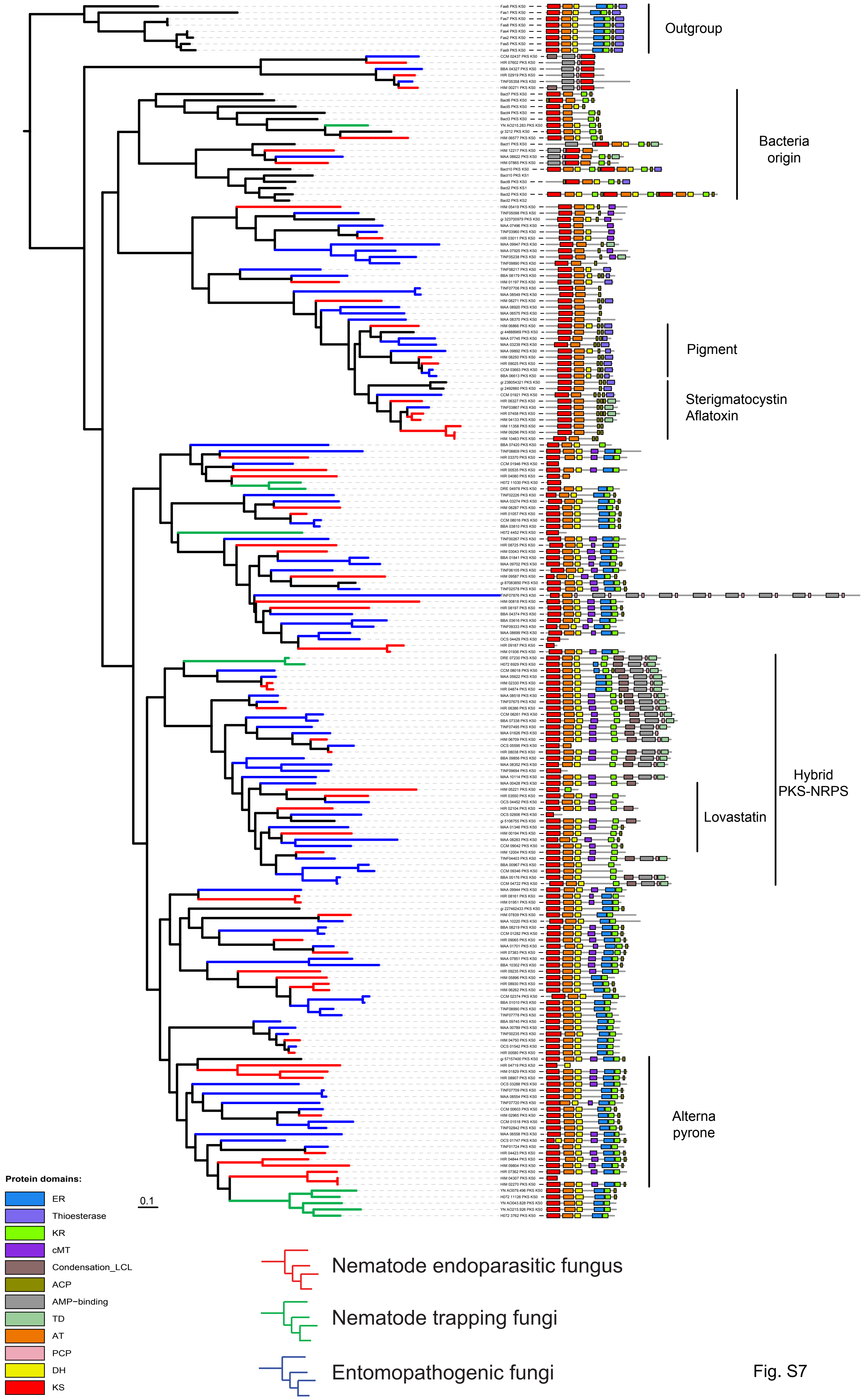
Outgroup
Bacteria origin
Pigment
Sterigmatocystin
Aflatoxin
Hybrid PKS-NRPS
Lovastatin
Alterna pyrone
Protein domains:
ER
Thioesterase
KR
cMT
Condensation_LCL
ACP
AMP−binding
TD
AT
PCP
DH
KS
0.1
Nematode endoparasitic fungus
Nematode trapping fungi
Entomopathogenic fungi

Fig. S7

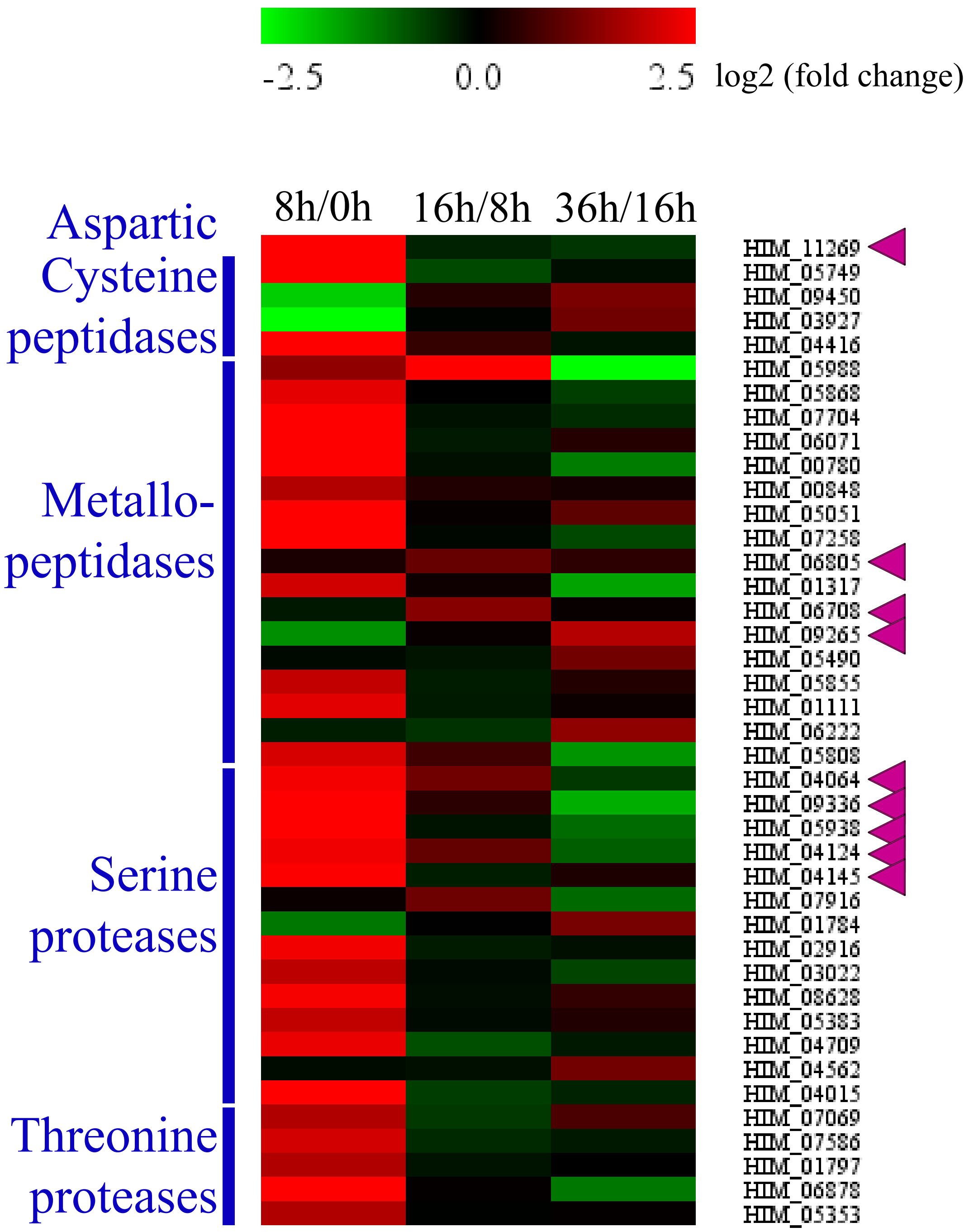
-2.5
0.0
2.5
log2 (fold change)
8h/0h
16h/8h
36h/16h
Aspartic
Cysteine peptidases
Metallo-peptidases
Serine proteases
Threonine proteases
HIM_11269
HIM_05749
HIM_09450
HIM_03927
HIM_04416
HIM_05988
HIM_05868
HIM_07704
HIM_06071
HIM_00780
HIM_00848
HIM_05051
HIM_07258
HIM_06805
HIM_01317
HIM_06708
HIM_09265
HIM_05490
HIM_05855
HIM_01111
HIM_06222
HIM_05808
HIM_04064
HIM_09336
HIM_05938
HIM_04124
HIM_04145
HIM_07916
HIM_01784
HIM_02916
HIM_03022
HIM_08628
HIM_05383
HIM_04709
HIM_04562
HIM_04015
HIM_07069
HIM_07586
HIM_01797
HIM_06878
HIM_05353

Fig. S8

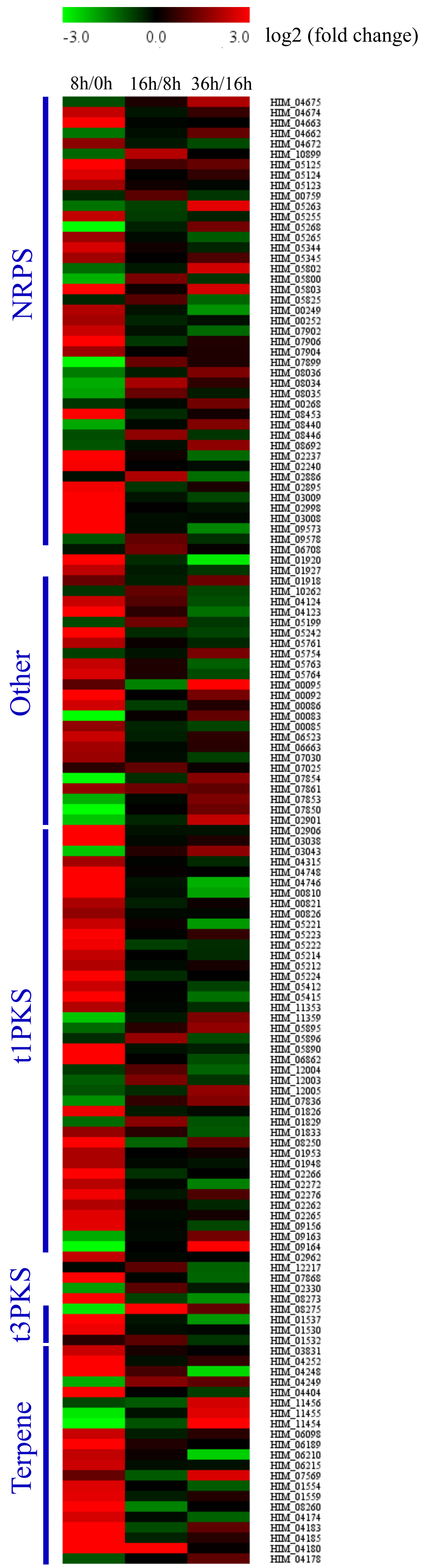

-3.0
0.0
3.0
log2 (fold change)
8h/0h
16h/8h
36h/16h
NRPS
Other
t1PKS
t3PKS
Terpene
HIM_04675
HIM_04674
HIM_04663
HIM_04662
HIM_04672
HIM_10899
HIM_05125
HIM_05124
HIM_05123
HIM_00759
HIM_05263
HIM_05255
HIM_05268
HIM_05265
HIM_05344
HIM_05345
HIM_05802
HIM_05800
HIM_05803
HIM_05825
HIM_00249
HIM_00252
HIM_07902
HIM_07906
HIM_07904
HIM_07899
HIM_08036
HIM_08034
HIM_08035
HIM_00268
HIM_08453
HIM_08440
HIM_08446
HIM_08692
HIM_02237
HIM_02240
HIM_02886
HIM_02895
HIM_03009
HIM_02998
HIM_03008
HIM_09573
HIM_09578
HIM_06708
HIM_01920
HIM_01927
HIM_01918
HIM_10262
HIM_04124
HIM_04123
HIM_05199
HIM_05242
HIM_05761
HIM_05754
HIM_05763
HIM_05764
HIM_00095
HIM_00092
HIM_00086
HIM_00083
HIM_00085
HIM_06523
HIM_06663
HIM_07030
HIM_07025
HIM_07854
HIM_07861
HIM_07853
HIM_07850
HIM_02901
HIM_02906
HIM_03038
HIM_03043
HIM_04315
HIM_04748
HIM_04746
HIM_00810
HIM_00821
HIM_00826
HIM_05221
HIM_05223
HIM_05222
HIM_05214
HIM_05212
HIM_05224
HIM_05412
HIM_05415
HIM_11353
HIM_11359
HIM_05895
HIM_05896
HIM_05890
HIM_06862
HIM_12004
HIM_12003
HIM_12005
HIM_07836
HIM_01826
HIM_01829
HIM_01833
HIM_08250
HIM_01953
HIM_01948
HIM_02266
HIM_02272
HIM_02276
HIM_02262
HIM_02265
HIM_09156
HIM_09163
HIM_09164
HIM_02962
HIM_12217
HIM_07868
HIM_02330
HIM_08273
HIM_08275
HIM_01537
HIM_01530
HIM_01532
HIM_03831
HIM_04252
HIM_04248
HIM_04249
HIM_04404
HIM_11456
HIM_11455
HIM_11454
HIM_06098
HIM_06189
HIM_06210
HIM_06215
HIM_07569
HIM_01554
HIM_01559
HIM_08260
HIM_04174
HIM_04183
HIM_04185
HIM_04180
HIM_04178

Fig. S9

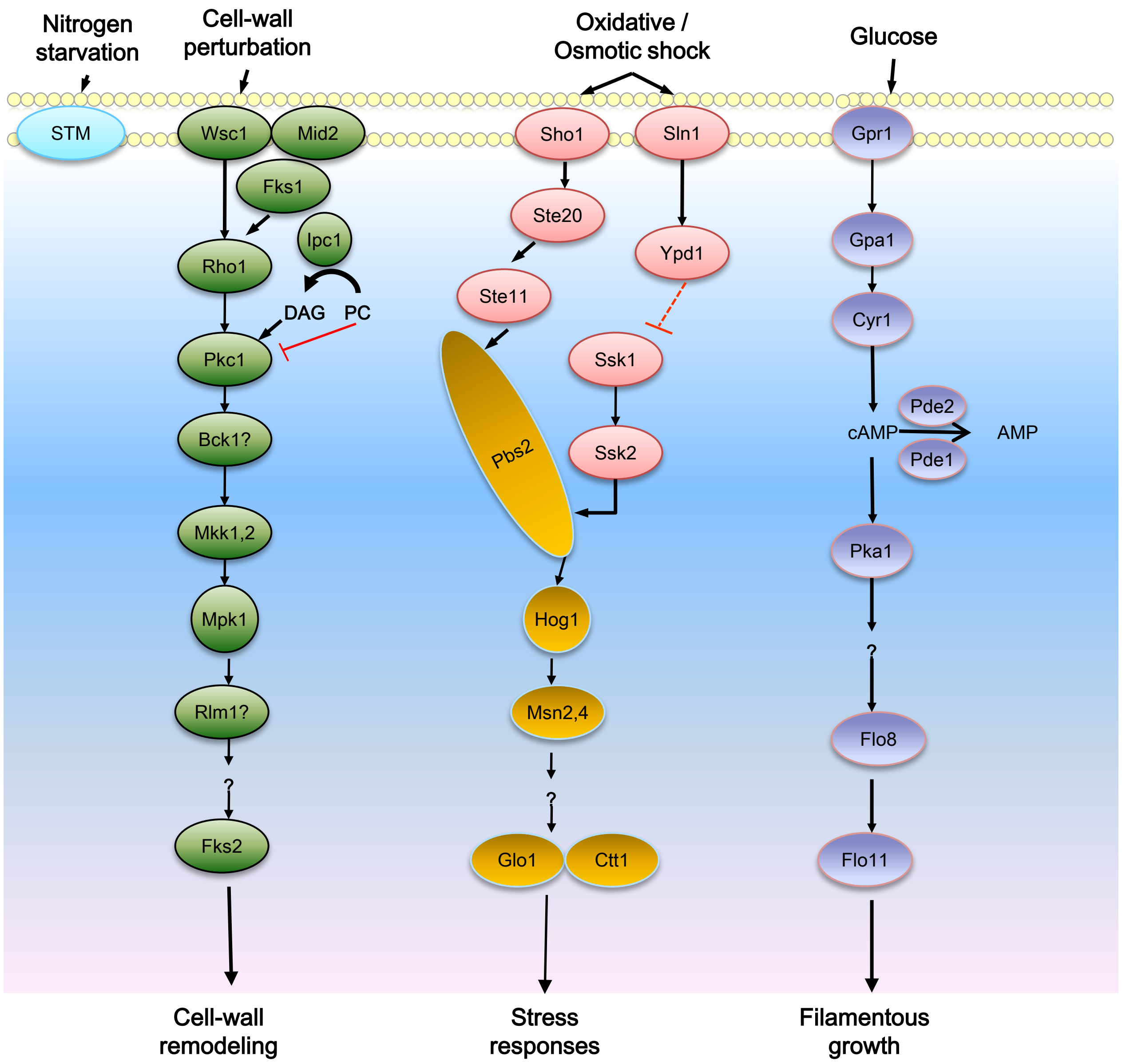
Nitrogen starvation
Cell-wall perturbation
Oxidative / Osmotic shock
Glucose
STM
Wsc1
Mid2
Fks1
Ipc1
Rho1
DAG
PC
Pkc1
Bck1?
Mkk1,2
Mpk1
Rlm1?
?
Fks2
Sho1
Sln1
Ste20
Ypd1
Ste11
Ssk1
Pbs2
Ssk2
Hog1
Msn2,4
?
Glo1
Ctt1
Gpr1
Gpa1
Cyr1
Pde2
cAMP
AMP
Pde1
Pka1
?
Flo8
Flo11
Cell-wall remodeling
Stress responses
Filamentous growth

Fig. S10
