# Supplementary material for: Comparative Genomics and Transcriptomics Analyses Reveal Divergent Lifestyle Features of Nematode Endoparasitic Fungus Hirsutella minnesotensis
Source: Genome Biol Evol. 2014 Oct 30;6(11):3077–93. doi: 10.1093/gbe/evu241 (PMC4255773; doi:10.1093/gbe/evu241)
Supplement: Supplementary Data [file supp_evu241_New_Microsoft_Office_Word_Document.docx]

**Supplementary figure legends**

**Fig. S1. The diversity and distribution of class I and class II transposable elements in *H. minnesotensis* genome.** The TEs were identified using the REPET pipeline. 535 consensus sequences were firstly predicted using the TEdenovo pipeline. Based on these consensus sequences, the number of TE occurrences and the coverage of genome sequences were identified with REPET TEannot pipeline. The superfamily structures were modified from Wicker et al. (Nature Reviews Genetics, 2007(8): 973-982). Abbreviations: LTR, long terminal repeat retrotransposons; LINE, long interspersed elements; SINE, short interspersed elements; TIR, terminal inverted repeats.

**Fig. S2. Distribution of genes, DNA transposons, retrotransposons and no category TEs on the five selected scaffolds of *H. minnesotensis*.**

**Fig. S3. Estimation of RIP for *H. minnesotensis* genome.** Average RIP index and the ratio of each type of di-nucleotide (ratio of frequency in TEs to that in non-TEs) in the genome are shown.

**Fig. S4. The RIP index (TpA/ApT) of genes as a function of the distance from a transposable element.** The RIP index is highest near the transposable elements and levels off after approximately 1000 bp, signifying that these regions are subjected to repeat induced point mutations.

**Fig. S5. Phylogenomic relationships and divergence time of *H. minnesotensis* and other 14 Ascomycota species.**

**Fig. S6. Parasitism of *H. minnesotensis* on soybean cyst nematodes treated with lectins (A) and mixtures (B).** Mix-conA means mixture of three lectins including RCA, HPA and MBL but except conA and other versa. A statistical *P* value of less than 0.05 between each two treatments determined using the unpaired two-tailed Student’s *t*-test was considered to be significant and labeled with different lowercases.

**Fig. S7. Phylogenetic tree of nematophagous and insect fungal PKS genes containing conserved KS domains annotated by Pfam.**

**Fig. S8. Heatmaps of protease gene expression during parasitism of *H. minnesotensis* on nematodes.** Heatmaps shows the most highly expressed and significantly regulated genes (log2 fold change >2, *P* < 0.05). The triangular symbols indicate proteins with a signal peptide.

**Fig. S9. Heatmaps of secondary metabolite gene expression during parasitism of *H. minnesotensis* on nematodes.** Heatmaps shows the most highly expressed and significantly regulated genes (log2 fold change >2, *P* < 0.05).

**Fig. S10. Putative pathways involved in nematode endoparasitism.**
